# New Capoamycin-Type Antibiotics and Polyene Acids from Marine *Streptomyces fradiae* PTZ0025

**DOI:** 10.3390/md10112388

**Published:** 2012-10-29

**Authors:** Wenxiu Xin, Xuewei Ye, Siran Yu, Xiao-Yuan Lian, Zhizhen Zhang

**Affiliations:** 1 College of Pharmaceutical Sciences, Zhejiang University, Hangzhou 310058, China; Email: xiaoxindream@163.com; 2 Department of Ocean Science and Engineering, Zhejiang University, Hangzhou 310058, China; Email: 531023833@qq.com (X.Y.); yusiran_321@qq.com (S.Y.)

**Keywords:** marine *Streptomyces fradiae*, fradimycins A and B, fradic acids A and B, antibacterial and antitumor activities

## Abstract

Capoamycin-type antibiotics (**2**–**5**) and polyene acids (**6**, **7**) were isolated from marine *Streptomyces fradiae* strain PTZ0025. Their structures were established by extensive nuclear magnetic resonance (NMR) and high resolution electron spray ionization mass spectroscopy (HRESIMS) analyses and chemical degradation. Compounds **3**, **4**, **6**, **7** were found to be new and named as fradimycins A (**3**) and B (**4**), and fradic acids A (**6**) and B (**7**). Compounds **3**–**5** showed *in vitro* antimicrobial activity against *Staphylococcus aureus* with a minimal inhibitory concentration (MIC) of 2.0 to 6.0 μg/mL. Interestingly, Compounds **3**–**5** also significantly inhibited cell growth of colon cancer and glioma with IC_50_ values ranging from 0.13 to 6.46 μM. Fradimycin B (**4**), the most active compound, was further determined to arrest cell cycle and induce apoptosis in tumor cells. The results indicated that fradimycin B (**4**) arrested the cell cycle at the G_0_/G_1_ phase and induced apoptosis and necrosis in colon cancer and glioma cells. Taken together, the results demonstrated that the marine natural products **3**–**5**, particularly fradimycin B (**4**), possessed potent antimicrobial and antitumor activities.

## 1. Introduction

**Figure 1:**
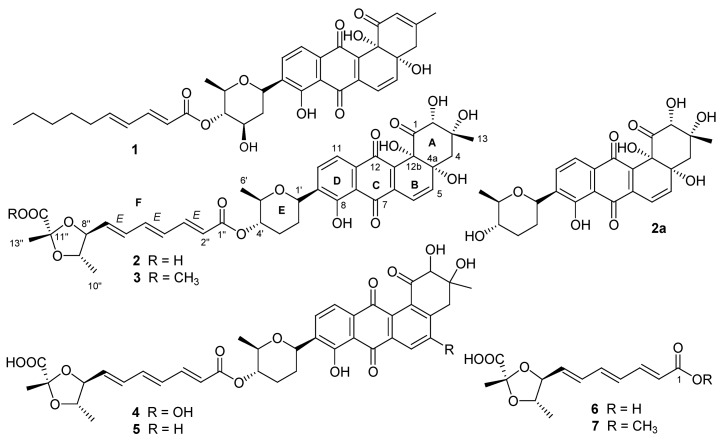
Structures of Compounds **1**–**7**.

Capoamycin (**1**, Figure 1) was originally isolated from the culture broth of soil *Streptomyces capoamus* strain No. 23–41 [1]. Its characteristic structure consists of a modified benz[*a*]anthraquinone chromophore, a deoxysugar unit and a long chain polyene acid. Capoamycin has been reported to inhibit the growth of gram-positive bacteria, yeasts and fungi, induce differentiation of mouse myeloid leukemia cells, and prolong the survival periods of mice bearing Ehrich ascites carcinoma [1,2]. The capoamycin-related antibiotics included dioxamycin (**2**) from soil *Streptomyces xantholiticus* strain MH406-SF1 [3] and MK844-mF10 (**5**), which was recently discovered from soil *Streptomyces* sp. MK844-mF10 [4]. MK844-mF10 had activities against multidrug-resistant *Staphylococcus aureus* and vancomycin-intermediated *Staphylococcus aureus* and inhibited the activities of *Bacillus subtilis* YycG histidine kinase, *Streptococcus mutans* Vick histidine kinase, and *Erwinia carotovora* PehS histidine kinase [4].

During the course of our ongoing program for the discovery of novel bioactive substances from marine microorganisms, a crude extract prepared from the pellet of the cultured *Streptomyces fradiae* strain PTZ00025 isolated from marine sediments was shown to be active against *Staphylococcus aureus*. Bioassay-guided isolation of this active crude resulted in the isolation and identification of the known antibiotics dioxamycin (**2**) and MK844-mF10 (**5**), new compounds of fradimycins A (**3**) and B (**4**) and fradic acids A (**6**) and B (**7**) (Figure 1). Compounds **3–5** showed antibacterial and antitumor activities. In this study, the isolation and structure elucidation of these new compounds and their antibacterial and antitumor properties are described.

## 2. Results and Discussion

The antibiotic-producing strain PTZ0025 was isolated from marine sediments. The taxonomic identity of the isolated strain was determined by 16S rDNA sequence analysis. The top sequence match as of August 2012, using Basic Local Alignment Search Tool (BLAST) (nucleotide sequence comparison) and the GenBank database was a 99.0% sequence similarity to *Streptomyces fradiae* strain CO-4 (GenBank accession number JF915304.1).

An acetone extract prepared from the pellet of the cultured broth of this isolated strain had activity against *Staphylococcus aureus*. This active crude was fractionated by Diaion HP-20 column chromatography, following by repeated high-performance liquid chromatography (HPLC) purification (supplementary Figure S43) to furnish Compounds **2–7**.

Compound **2** was proven to be identical to dioxamycin by comparison of its physico-chemical properties and spectral data with the reported values [3]. Dioxamycin has reported to have activities *in vitro* against gram-positive bacteria and some tumor cells [3].

**Figure 2:**
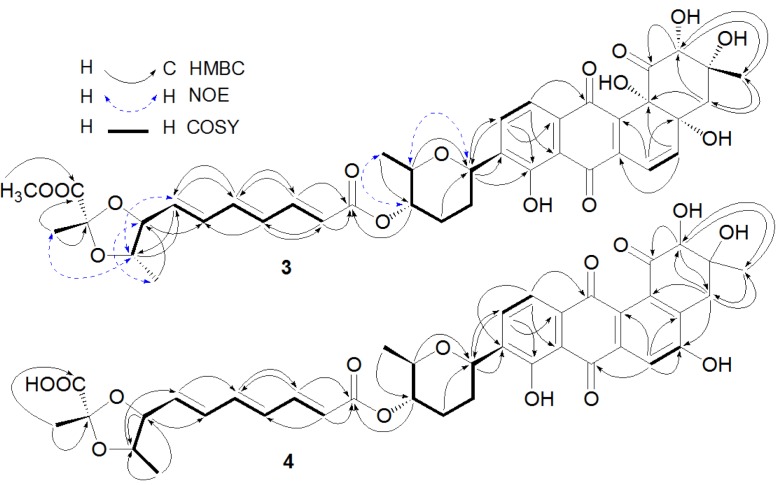
Key heteronuclear multiple bond correlation (HMBC), nuclear Overhauser effect (NOE), and correlation spectroscopy (COSY) correlations of Compounds **3** and **4**.

Compound **3** was obtained as an orange amorphous powder and had a molecular formula of C_39_H_42_O_15_ deduced from its high resolution electron spray ionization mass spectroscopy (HRESIMS) *m*/*z* at 751.2673 [M + H]^+^ and 773.2463 [M + Na]^+^, 14 mass units higher than that of **2**. Compound **3** exhibited similar UV absorption to **2**. The ^13^C and ^1^H nuclear magnetic resonance (NMR) data of **3** (Table 1 and Table 2) were also very close to those of **2** with the exception of additional signals at δ_H_ 3.76 (3H, s) and δ_C_ 53.1 for a methoxyl in **3**. An upfield chemical shift (∆δ −1.3 ppm) for C-12″ between **3** and **2** suggested the methoxyl at C-12″. A key heteronuclear multiple bond correlation (HMBC) of δ_H_ 3.76 (OCH_3_) to δ_C_ 172.6 (C-12″) further supported the above-mentioned conclusion. The *trans*-coupling constants of 15.1 Hz (H-2″ and H-3″), 14.8 Hz (H-4″ and H-5″), and 15.0 Hz (H-6″ and H-7″) determined the double bond geometries of 2″*E*, 4″*E* and 6″*E* in **3**. In the rotating frame nuclear Overhauser effect spectroscopy (ROESY) spectrum of **3**, the key nuclear Overhauser effect (NOE) correlations (Figure 2) of H-1′ with H-5′, H-4′ with H-6′, H-7″ with H-9″, H-8″ with H-10″, and H-9″ with H-13″ suggested that **3** had the assigned relative stereochemistry at C-1′, C-4′, C-5′, C-8″, C-9″, and C-11″, which were the same as their counterparts in **2** [3]. After alkaline hydrolysis, both **3** and **2** furnished a same product **2a**, confirmed by their UV, [α]_D_, and co-HPLC analysis, suggesting that **3** and **2** also possessed the same stereochemistry at C-2, C-3, C-4a and C-12b. The ^1^H and ^13^C NMR signals of **3** were assigned by a combination of ^1^H, ^13^C, heteronuclear multiple quantum correlation (HMQC), HMBC, and ROESY NMR spectroscopic analysis, and by comparison with the NMR data of **2**. The structure of **3** was thus established as fradimycin A, a new marine natural product.

Compound **4** was obtained as an orange amorphous powder and had a molecular formula of C_38_H_38_O_1__4_ as determined by its HRESIMS ions at *m*/*z* 719.2408 [M + H]^+^ and 741.2247 [M + Na]^+^. Its UV spectrum displayed maxima absorption at 294 and 427 nm. The ^13^C NMR data (Table 1) of **4** showed 38 signals, of which six were assigned to the 2,3-dideoxysugar moiety (ring E), 13 to the polyene acid unit (part F), and the remaining 19 to the anthraquinone chromophore (rings A–D). Detailed analysis of the NMR data of **4** and **2** concluded that both compounds shared identical substructures of ring E and part F.

**Table 1:** ^13^C nuclear magnetic resonance (NMR) data for compounds **2**–**5** (125 MHz, in dimethyl sulfoxide (DMSO)-*d*_6_) ^a^.

Position	2 ^b^	2	3 ^b^	3	4 ^b^	4	5
1	206.6	205.1	206.6	205.0	200.5	198.3	197.9
2	82.9	81.4	83.0	81.4	82.7	79.8	80.1
3	77.6 ^c^	75.4	77.7 ^c^	75.4	76.5	73.3	74.5
4	45.5	44.7	45.7	44.5	38.1	35.8	40.9
4a	77.5 ^c^	76.1	77.6 ^c^	76.1	129.2	127.5	148.8
5	147.8	147.5	147.8	147.5	161.4	159.5	134.1
6	117.7	115.7	117.7	115.6	113.8	112.0	128.7
6a	139.5 ^d^	137.5 ^c^	139.5	137.4 ^c^	138.0 ^c^	137.2 ^c^	133.8 ^c^
7	189.7	188.5	189.9	188.4	189.5	187.8	187.6
7a	115.3	114.1	115.5	114.0	116.3	114.8	114.8
8	158.9	156.9	159.1	156.9	159.4	157.3	157.4
9	139.8 ^d^	137.6 ^c^	140.0 ^d^	137.5 ^c^	138.3 ^c^	137.3 ^c^	136.4 ^d^
10	134.6	133.3	134.6	133.2	134.7	133.1 ^d^	133.4 ^c^
11	120.4	118.7	120.2	118.6	120.1	118.5	118.6
11a	131.9	130.5	132.0	130.4	135.5	134.1 ^d^	133.0 ^c^
12	183.8	182.1	183.9	182.0	183.1	181.0	182.3
12a	139.9 ^d^	138.7	139.9 ^d^	138.6	137.0 ^d^	138.0 ^c^	136.5 ^d^
12b	78.4	76.8	78.3	76.7	136.9 ^d^	136.1 ^c^	134.8 ^c^
13	22.0	21.8	21.9	21.8	22.7	24.9	24.6
1′	74.6	72.5	74.7	72.4	74.7	72.4	72.5
2′	32.6	31.0	32.7	30.9	32.7	31.0	31.0
3′	30.7	29.2	30.8	29.1	30.8	29.3	29.2
4′	74.8	72.9	74.9	72.8	74.9	72.9	72.9
5′	77.2	75.3	77.1	75.3	77.0	75.3	75.4
6′	18.9	18.3	18.8	18.2	18.9	18.2	18.2
1″	168.0	165.6	167.9	165.4	167.9	165.5	165.5
2″	122.5	121.2	122.6	121.3	122.6	121.5	121.2
3″	146.3	144.8	146.3	144.7	146.3	144.7	144.7
4″	132.6	131.2	132.7	131.2	132.6	131.1	131.1
5″	141.2	140.0	141.1	139.8	141.2	139.9	140.0
6″	134.8	133.2 ^d^	134.9	133.3	134.8	134.5 ^d^	132.2 ^c^
7″	134.0	133.1 ^d^	133.9	132.7	134.1	133.1 ^d^	133.1 ^c^
8″	85.9	83.9	86.1	83.9	86.0	83.8	83.9
9″	79.3	77.1	79.4	77.2	79.4	77.2	77.1
10″	17.0	16.4	16.8	16.3	16.9	16.3	16.4
11″	106.3	104.5	106.4	104.4	106.4	104.4	104.5
12″	173.9	171.4	172.6	170.1	174.0	171.3	171.4
13″	23.5	22.9	23.5	22.9	23.5	22.9	22.9
OCH_3_			53.1	52.3			

^a^ The assignment was based on ^1^H, ^13^C NMR, ^1^H-^1^H COSY, HMQC, and HMBC spectra. ^b^ Data were recorded in CD_3_OD. ^c, d^ Data with the same letter in each column may be interchangeable.

**Table 2:** ^1^H NMR data for Compounds **2–5** (500 MHz, in DMSO-*d*_6_) ^a^.

H	2 ^b^	2	3 ^b^	3	4 ^b^	4	5
2	4.31, s	4.13, s	4.28, s	4.13, s	4.56, s	4.02, s	4.04, s
4	1.88, d	1.80, d	1.86, d	1.80, d	2.98, d	2.88, s	3.00, d
	(14.9);	(14.7);	(14.8);	(14.7);	(18.0);		(17.3);
	2.15, d	1.91, d	2.13, d	1.91, d	3.24, d		3.20, d
	(14.9)	(14.7)	(14.8);	(14.7)	(18.0)		(17.3)
5	6.41, d (9.8)	6.38, d (9.7)	6.40, d (9.8)	6.38, d (9.7)	–	–	7.71, d (8.0)
6	6.82, d (9.8)	6.68, d (9.7)	6.85, d (9.8)	6.68, d (9.7)	7.71, s	7.66, s	8.23, d (8.0)
10	7.89, d (7.8)	7.85, d (7.8)	7.89, d (7.8)	7.86, d (7.8)	7.87, d (7.8)	7.85, d (7.8)	7.88, d (7.8)
11	7.60, d (7.8)	7.54, d (7.8)	7.60, d (7.8)	7.54, d (7.8)	7.58, d (7.8)	7.54, d (7.8)	7.57, d (7.8)
13	1.22, s	1.03, s	1.22, s	1.03, s	1.18, s	1.20, s	1.21, s
1′	4.83 ^c^	4.83, d (11.2)	4.88, d (12.1)	4.84, d (11.1)	4.86 ^c^	4.84, d (12.2)	4.86, d (11.0)
2′	1.50, m;	1.51, m;	1.57, m;	1.51, m;	1.56, m;	1.51, m;	1.51, m;
	2.24, m	2.13, m	2.25, m	2.13, m	2.27, m	2.14, m	2.15, m
3′	1.75, m;	1.72, m;	1.76, m;	1.75, m;	1.77, m;	1.73, m;	1.75, m;
	2.22, m	2.15, m	2.23, m	2.15, m	2.23, m	2.15, m	2.17, m
4′	4.57, m	4.51, m	4.61, m	4.53, m	4.60, m	4.54, m	4.56, m
5′	3.69, m	3.70, m	3.70, m	3.72, m	3.70, m	3.72, m	3.73, m
6′	1.26, d (6.1)	1.17, d (6.7)	1.26, d (6.1)	1.17, d (6.8)	1.27, d (6.1)	1.21, d (6.2)	1.20, d (6.1)
2″	5.97, d (15.3)	6.04, d (15.2)	5.98, d (15.3)	6.05, d (15.1)	5.98, d (15.1)	6.05, d (15.1)	6.05, d (15.2)
3″	7.32, dd	7.29, dd	7.34, dd	7.30, dd	7.34, dd	7.30, dd	7.30, dd
	(11.2, 15.3)	(11.3, 15.2)	(11.2, 15.3)	(10.2, 15.1)	(11.3, 15.1)	(11.2, 15.1)	(11.5, 15.2)
4″	6.47, dd	6.51, dd	6.48, dd	6.52, dd	6.48, dd	6.52, dd	6.52, dd
	(11.2, 14.9)	(11.3, 14.9)	(11.2, 15.0)	(10.2, 14.8)	(11.3, 15.0)	(11.2, 14.7)	(11.5, 15.0)
5″	6.69, dd	6.78, dd	6.69, dd	6.78, dd	6.69, dd	6.78, dd	6.78, dd
	(10.9, 14.9)	(10.9, 14.9)	(10.8, 15.0)	(10.2, 14.8)	(11.0, 15.0)	(11.2, 14.7)	(11.0, 15.0)
6″	6.53, dd	6.51, dd	6.53, dd	6.52, dd	6.53, dd	6.52, dd	6.55, dd
	(10.9, 15.1)	(10.9, 15.3)	(10.8, 15.2)	(10.2, 15.0)	(11.0, 15.2)	(11.2, 15.0)	(11.0, 15.3)
7″	5.90, dd	5.92, dd	5.90, dd	5.91, dd	5.91, dd	5.93, dd	5.93, dd
	(7.5, 15.1)	(7.5, 15.3)	(7.5, 15.2)	(7.6, 15.0)	(7.4, 15.2)	(7.3, 15.0)	(7.5, 15.3)
8″	4.22, t	4.23, t	4.27, t	4.23, t	4.27, t	4.24, t	4.25, t
	(7.5, 7.9)	(7.5, 7.9)	(7.5, 7.9)	(7.6, 7.6)	(7.4, 8.3)	(7.3, 8.1)	(7.5, 7.9)
9″	3.83, m	3.78, m	3.83, m	3.83, m	3.83, m	3.80, m	3.79, m
10″	1.27, d (6.1)	1.18, d (6.7)	1.26, d (6.8)	1.19, d (6.2)	1.28, d (6.1)	1.19, d (6.0)	1.19, d (6.1)
13″	1.55, s	1.46, s	1.55, s	1.50, s	1.55, s	1.46, s	1.45, s
5-OH	–	–	–	–	–	11.4, s	–
8-OH	12.3, s	12.2, s	–	12.2, s	–	12.6, s	12.6, s
OCH_3_	–	–	3.76, s	3.70, s	–	–	–

^a^ The assignment was based on ^1^H, ^13^C NMR, ^1^H-^1^H COSY, HMQC and HMBC spectra. ^b^ Data were recorded in CD_3_OD. ^c^ This signal overlapped the solvent signal.

The anthraquinone chromophore in **4** was constructed by NMR spectroscopic analysis and by comparison of its NMR data with those of its counterpart in **2**. In the ^1^H NMR spectrum (Table 2, in DMSO-*d*_6_) of the anthraquinone part in **4**, three aromatic proton signals at δ 7.66 (1H, s), δ 7.85 (1H, d, 7.8 Hz), and δ 7.54 (1H, d, 7.8 Hz) were attributed to H-6, H-10, and H-11, respectively. The three singlet signals at δ 4.02 (1H, s), 2.88 (2H, s), and 1.20 (3H, s) were assigned to H-2, H-4, and H-13, respectively. In addition, two singlets at δ 11.4 (1H, s) for 5-OH and δ 12.6 (1H, s) for 8-OH were also observed in the ^1^H NMR spectrum of **4**. The ^13^C NMR spectrum of **4** (in DMSO-*d*_6_) displayed signals at δ 127.5 for C-4a and δ 136.1 for C-12b, instead of two signals at δ 76.1 and δ 76.8 for the two oxyquaternary carbons (C-4a and C-12b) as in **2**. Above evidence suggested the existence of a double bond between C-4a and C-12b in **4**, instead of the two hydroxyl groups at C-4a and C-4b as in **2**. The double bond geometries for the polyene acid in **4** were assigned as 2″*E*, 4″*E* and 6″*E* based on the *trans*-coupling constants (Table 2). Unfortunately, ROESY experiment for **4** did not provide useful NOE information due to its limited amount for the assignment of the relative stereochemistry at C-2 and C-3 in **4**. However, the relative stereochemistry at C-1′, C-4′, C-5′, C-8″, C-9″, and C-11″ in **4** was proposed to be the same as those in **2** because the NMR values for ring E and part F between **4** and **2** were almost the same (Table 1 and Table 2). After alkaline hydrolysis, both **4** and **2** produced the same compound 6 as confirmed by UV, [α]_D_, and co-HPLC analysis, further supporting the assigned stereochemistry at C-8″, C-9″, and C-11″ in **4**. The complete assignments of ^1^H and ^13^C NMR data of **4** were made by HMQC and HMBC correlations (Figure 2). The structure of 4 was determined as fradimycin B, a new compound.

The molecular formula of C_38_H_38_O_13_ for **5** was determined by HRESIMS and indicated that it had one less oxygen atom when compared with **4**. Its UV spectrum and [α]_D_ value were very similar to those of **4**. Examination of the NMR data of **5** and **4** showed that 5 differed from **4** only in the substituent at C-5 of ring B. In the NMR spectra, the signals at δ_C_ 159.5 (C, C-5), δ_C_ 112.0 (CH, C-6), and δ_H_ 7.66 (1H, s, H-6) in **4** were replaced by the signals at δ_C_ 134.1 (CH, C-5), δ_C_ 128.7 (CH, C-6), δ_H_ 7.71 (1H, d, *J* = 8.0 Hz, H-5), and δ_H_ 8.23 (1H, d, *J* = 8.0 Hz, H-6) in **5**. The above information demonstrated that **5** was related to **4** without the hydroxyl group at C-5. Accordingly, the structure of **5** was assigned as MK844-mF10 that was recently isolated from *Streptomyces* sp. MK844-mF10 [4]. 

Compounds **6** and **7** had molecular formulae of C_13_H_16_O_6_ and C_14_H_18_O_6_, respectively, deduced from their HRESIMS and ^13^C NMR data. Both compounds contained characteristic NMR signals of two methyls, two oxymethines, one oxyquaternary carbon, six olefinic carbons, and two carbonyls. The ^13^C and ^1^H chemical shifts of **6** (Table 3) were very close to those of the polyene acid unit (part F) in **2** (Table 1 and Table 2, in DMSO-*d_6_*). The ^13^C chemical shift differences for C-1 (∆δ +2.0 ppm), C-2 (∆δ +1.6 ppm), and C-3 (∆δ −1.0 ppm) between **6** and the part F of **2** accounted for the existence of a free carboxylic acid at C-1 in **6**. The assigned structure of **6** was also confirmed by its [α]_D_ and co-HPLC analysis with an authentic sample, the polyene acid obtained from a product of alkaline hydrolysis of **2**. The ^1^H and ^13^C NMR signals of **6** were assigned using HMQC and HMBC correlations (Table 3). Therefore, the structure of **6** was assigned as fradic acid A.

**Table 3:** NMR data for fradic acids A (**6**) and B (**7**) (in DMSO-*d*_6_).

position	6			7	
	δ_C_	δ_H_ ( *J* in Hz)	HMBC	δ_C_	δ_H_ ( *J* in Hz)
1	167.6	–	–	165.5	–
2	122.8	5.91, d (15.1)	C_1_, C_4_	121.3	6.05, d (15.2)
3	143.8	7.18, dd (11.3, 15.1)	C_1_, C_2_, C_4_, C_5_	144.7	7.30, dd (11.0, 15.2)
4	131.5	6.48, dd (11.3, 14.8)	C_2_, C_3_, C_5_, C_6_	131.2	6.52, dd (11.0, 14.8)
5	139.0	6.69, dd (10.8, 14.8)	C_3_, C_7_	139.8	6.78, dd (11.0, 14.8)
6	133.3	6.51, dd (10.8, 15.2)	C_4_, C_5_, C_7_, C_8_	133.3	6.52, dd (11.0, 15.1)
7	132.5	5.89, dd (7.5, 15.2)	C_5_, C_8_, C_9_	132.7	5.93, dd (7.5, 15.1)
8	84.0	4.22, t (7.5)	C_6_, C_9_, C_10_	83.9	4.23, t (7.5)
9	77.1	3.78, m	C_7_, C_8_	77.2	3.83, m
10	16.4	1.18, d (6.1)	C_8_, C_9_	16.3	1.19, d (6.2)
11	104.5	–	–	104.4	–
12	171.2	–	–	171.1	–
13	22.9	1.45, s	C_11_, C_12_	22.9	1.50, s
OCH_3_	–	–	–	52.3	3.70, s

The NMR data of **7** were almost superimposable on those of **6** with the exception of additional signals at δ_C_ 52.3 and δ_H_ 3.70 (3H, s) for a methoxyl. A chemical shift difference (Δδ −2.1 ppm) for C-1 (δ 165.5) of **7** when compared to C-1 (δ 167.6) of **6** indicated that the methoxyl was linked to C-1 position. Both **7** and **6** had very similar UV spectra and [α]_D_ values. Therefore, the structure of **7**, a new compound, was established as fradic acid B.

It was possible that the isolates **3**, **6**, and **7** were artifacts during the work-up isolation. In order to exclude this possibility, an ethanol extract prepared from the pellet of the isolate strain PTZ0025 was analyzed by co-HPLC with authentic samples using acetonitrile/water without acid as flow phase. The results indicated that compounds **3**, **6**, and **7** as new natural products were detected in the original extract (supplementary Figure S43).

Compounds **3**–**7** were assayed *in vitro* for their antimicrobial activity against *Staphylococcus aureus*. The results showed that compounds **3**–**5** were active against *Staphylococcus aureus* with a MIC of 6.0, 2.0, and 4.0 μg/mL, respectively, whereas **6** and **7** were inactive.

**Figure 3:**
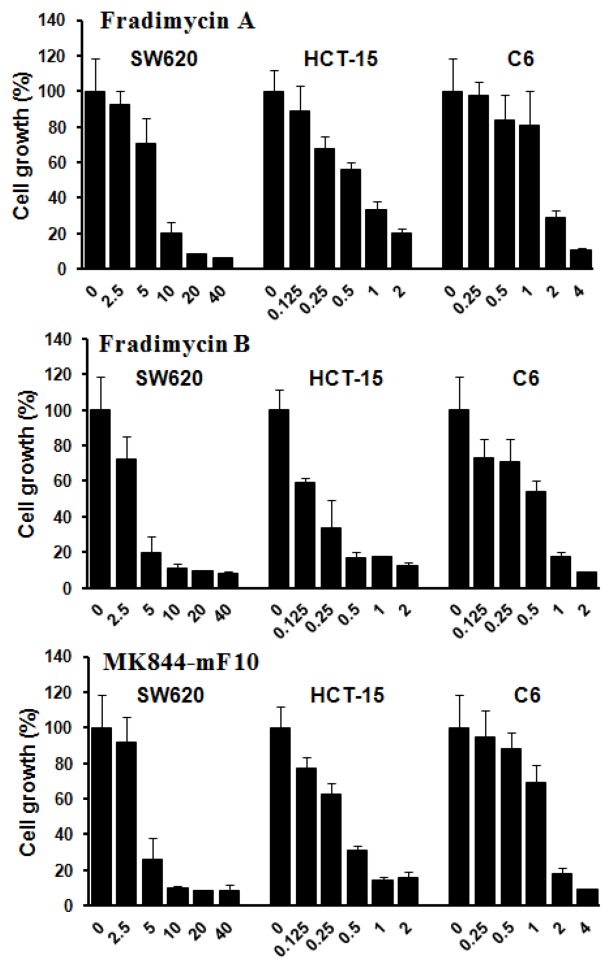
Tumor cell-growth inhibitory activity of fradimycins A (**3**), B (**4**) and MK844-mF10 (**5**). Human colon cancer HCT-15 and SW620 cells and rat glioma C6 cells were treated with fradimycins A, B and MK844-mF10 for 72 h at concentrations from 0.125 to 40 μM. Values are means ± S.D. from five independent experiments.

Compounds **3**–**5** were also tested *in vitro* for their cell-growth inhibitory activity against human colon cancer HCT-15 and SW620 cells as well as rat glioma C6 cells. All compounds showed dose-dependence activities of inhibiting growth of the tested tumor cells with IC_50_ values ranging from 0.13 ± 0.04 to 6.46 ± 1.44 μM (Figure 3 and Table 4).

**Table 4:** Inhibition of tumor cell-growth activity of Compounds **3–5**.

Cell lines	Human colon HCT-15			Human colon SW620			Rat glioma C6		
Compounds	3	4	5	3	4	5	3	4	5
IC_50_ (μM)	0.52 ± 0.11	0.13 ± 0.04	0.30 ± 0.07	6.46 ± 1.44	4.33 ± 1.56	4.39 ± 0.93	1.28 ± 0.37	0.47 ± 0.09	1.31 ± 0.32

**Figure 4:**
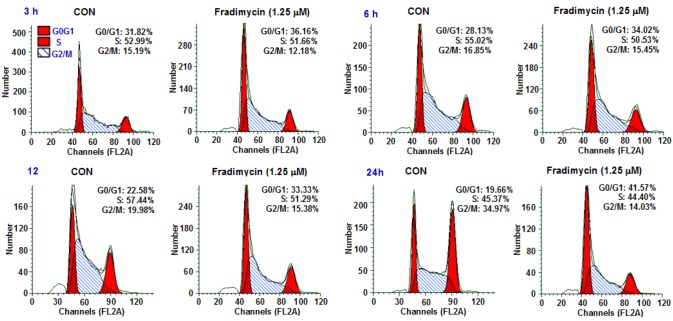
Fradimycin B (**4**) induced apoptosis and necrosis (cell cycle arrest) in HCT-15 cells. HCT-15 cells were incubated with fradimycins B (**4**, 1.25 μM) for 3 h, 6 h, 12 h and 24 h and then stained with propidium iodide (PI) and subjected to flow cytometric analysis for cell distribution at each phase of cell cycle. The percentage of cells at each stage of the cell cycle are shown. The cell population at the G_0_/G_1_ phase was significantly elevated (G_0_: cell cycle Gap 0 phase; G_1_: cell cycle Gap 1 phase; S: cell cycle synthesis phase; G_2_: cell cycle Gap 2 phase; M: cell cycle mitosis phase).

Cell cycle arrest and induction of apoptosis by fradimycin B (**4**) in HCT-15 cells was further investigated. To determine if an alteration of the cell cycle occurred following the treatment of fradimycin B (**4**), the DNA content was measured by flow cytometric analysis. The percentages of each phase in the cell cycle are represented in Figure 4 and Table 5. The proportion of cells in the G_0_/G_1_ phase of the cell cycle increased 4.34%, 5.89%, 10.75%, and 21.91% after 3 h, 6 h, 12 h, and 24 h exposure to 1.25 μM fradimycin B (**4**). The alteration occurring in the cell cycle suggested that fradimycin B (**4**) blocked HCT-15 cells at the G_0_/G_1_ phase.

**Table 5:** Time course analysis of cell cycle in fradimycin B (**4**) treated HCT-15 cells.

Treatment	Control			Fradimycin B (4, 1.25 μM)			Fradimycin B _(G0/G1)_^−^
	G_0_/G_1_	S	G_2_/M	G_0_/G_1_	S	G_2_/M	Control _(G0/G1)_
3 h	31.82%	52.99%	15.19%	36.16%	51.66%	12.18%	4.34%
6 h	28.13%	55.02%	16.85%	34.02%	50.53%	15.45%	5.89%
12 h	22.58%	57.44%	19.98%	33.33%	51.29%	15.38%	10.75%
24 h	19.66%	45.37%	34.97%	41.57%	44.40%	14.03%	21.91%

G_0_: cell cycle Gap 0 phase; G_1_: cell cycle Gap 1 phase; S: cell cycle synthesis phase; G_2_: cell cycle Gap 2 phase; M: cell cycle mitosis phase.

**Figure 5:**
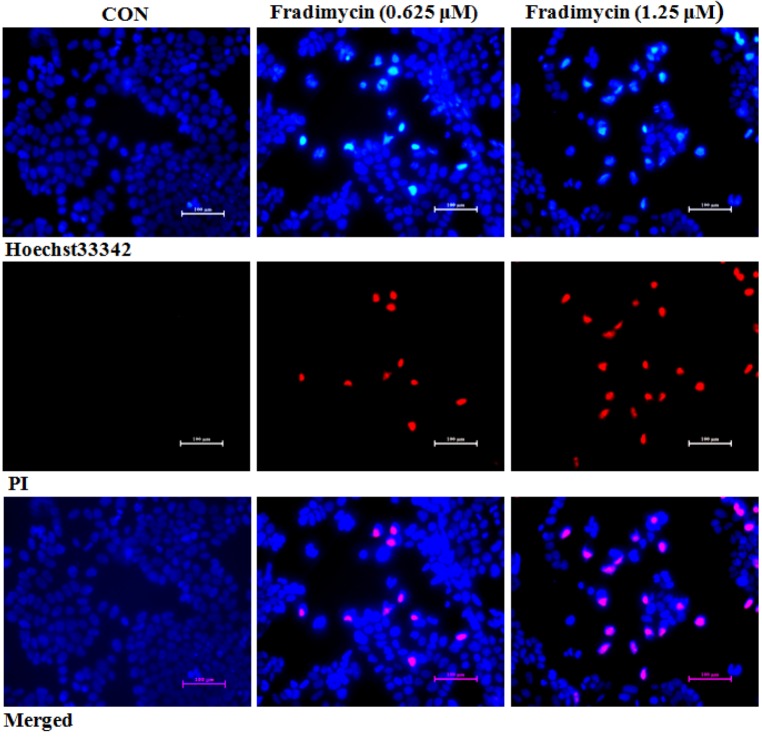
Fradimycin B (**4**) induced apoptosis and necrosis in HCT-15 cells. HCT-15 cells were treated with fradimycin B (**4**, 0.625 and 1.25 μM) for 72 h and then double stained with Hoechst 33342 and PI. Apoptotic cells showed bright blue nuclear condensation and necrotic cells displayed red fluorescence.

**Figure 6:**
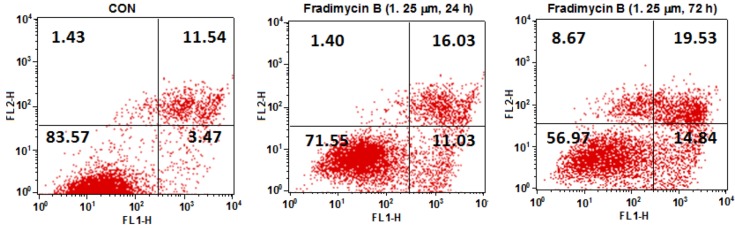
Dot-plot representing the double staining analysis with annixin V-FITC/PI in HCT-15 cells. HCT-15 cells were treated with fradimycin B (**4**, 1.25 μM) for 24 h and 72 h and were then stained with annexin-V FITC and PI and analyzed by flow cytometry. The fraction in the lower left represents normal cells, the fraction in the lower right represents early apoptotic cells, the fraction in the upper right represents late apoptotic cells, and the fraction in the upper left represents necrotic cells.

After 72 h of treatment, fradimycin B (**4**, 0.625 and 1.25 μM) induced apoptosis and necrosis in HCT-15, SW620 and C6 cells as detected by Hoechst 33342 and propidium iodide (PI) double staining (Figure 5 and Supplementary Figure S45 and Figure S46). In the cytometric analysis with annexin V-FITC/PI double staining, treatment with fradimycin B (**4**, 1.25 μM) in HCT-15 cells caused an increase of apoptotic and necrotic cells from 16.44% (control) to 28.46% (after 24 h treatment) and 43.04% (after 72 h treatment) (Figure 6).

## 3. Experimental Section

### 3.1. General Experimental Procedures

Optical rotations were measured on a JASCO DIP-370 digital polarimeter. UV spectra were recorded on a Shimadzu UV-2550 spectrometer. All NMR spectra were recorded on a Bruker 500 spectrometer at 500 MHz for ^1^H and 125MHz for ^13^C using standard pulse programs and acquisition parameters. Chemical shifts were expressed in δ (ppm) referring to the NMR solvent used. High-resolution ESIMS data were acquired on MicroMass Q-Tof-2 spectrometer or Agilent 6210 TOF LC/MS spectrometer. Diaion HP-20 was used for column chromatography. The separation and purification of compounds were conducted on the Agilent 1100 HPLC system using column A (Agilent Zorbax SB-C_18_ column, 250 × 9.4 mm, 5 μm, Supelco) and column B (ODS-2 Hypersil column, 250 × 4.6 mm, 5 μm, Thermo Scientific). Fluorescence microscope (Nikon SMZ1000) was used to detect apoptosis and necrosis of cells stained by Hoechst 33342 and propidium iodide (PI) double staining. Flow cytometry (Beckman Coulter, FC500MCL) was used for cell cycle assay and quantitative analysis of cell apoptosis.

Human colon cancer HCT-15 and SW620 cells and rat glioma C6 cells were purchased from the Cell Bank of the Chinese Academy of Sciences. Annexin V apoptosis detection kit was obtained from Invitrogen.

### 3.2. Isolation and Identification of *Streptomyces fradiae* Strain PTZ0025

The marine sediments (5 g) were air dried for 10 days in a 45 mL sterile centrifuge tube. The dried sample was diluted into 0.01 g/mL with seed broth (1.5% glucose, 1.5% glycerol, 1.5% malt extract, 2.5% yeast extract, 0.5% casamino acids, and 0.1% calcium carbonate), 200 μL of which was dispersed across a Bacto-agar plate and incubated at room temperature for 10 days. Bactria colonies were picked with sterile needles and purified by streaking them out onto Bacto-agar plates. After another seven days of growth at room temperature, the single colony (strain PTZ0025) that grew well was transferred onto Gause’s synthetic agar media. Working stocks were prepared on Gause’s synthetic agar slants and stored at 4 °C for further use.

The taxonomic identity of strain PTZ0025 was determined by 16S rDNA sequence analysis. The top sequence using BLAST (nucleotide sequence comparison) was compared to the GenBank database.

### 3.3. Fermentation of Strain PTZ0025

A colony of isolate PTZ0025 was homogenized using a sterile pestle in a 1.5 mL Eppendorf tube containing 300 μL of sterile sea water. The bacterial homogenate was transferred to a 250 mL Erlenmeyer flask containing 30 mL of seed broth and then incubated for seven days at 26 °C on a rotary shaker prior to production medium inoculation. Production was performed in 2.0 L tri-baffled flasks containing 500 mL aliquots of media (5.0 g glucose, 2.5 g yeast extract, 50 mg casamino acids, 3.0 g MgCl_2_·6H_2_O, 1.5 g CaCl_2_·2H_2_O, 100 mg K_2_SO_4_, 25 mg KH_2_PO_4_, and 50 mL sea water). The flasks were incubated for 10 days at 25–28 °C on a rotary shaker.

### 3.4. Isolation of Compounds **2–7**

The fermentation broth (2.0 L) of the isolate marine *Streptomyces fradiae* strain PTZ0025 was centrifuged at 8000 rpm for 20 min, and the pellet was extracted with acetone two times (each 1.0 L). The concentrated acetone extract (2.1 g), not the supernatant, was shown to be active. The active acetone extract was applied to a column of Diaion HP-20 (300 mL) washing in turn with 10% MeOH and 90% MeOH. The activity resided in 90% MeOH fraction. The dried active crude (500 mg) was redissolved in 1.5 mL of DMSO and then separated by HPLC using column A (UV detection: 295 nm) injected in multiple 100 μL runs with a gradient solvent system at a flow rate of 3.0 mL/min with 0.5 min for each collection from 3 to 27 min. Water and CH_3_CN containing 0.05% formic acid was employed as mobile phases A and B, respectively. The gradient procedure was 0–3.0 min with 10% B, 3.01–12.0 min with 40% B, 12.01–27.0 min with 65% B, 27.01–33.0 min with 100% B, and 33.01–36.0 with 10% B. After HPLC separation, fractions 8–9, 15–16, 29–30, 33–34, and 38 were lyophilized after the removal of CH_3_CN under reduced pressure to furnish fraction A (2.3 mg, *t_R_* 6.71 min), **7** (1.7 mg, *t_R_* 10.46 min), fraction B (3.6 mg *t_R_* 17.01 min), fraction C (4.6 mg *t_R_* 19.39 min), and **5** (0.9 mg, *t_R_* 21.30 min), respectively. Compound **6** (1.5 mg, *t_R_* 7.37 min) was purified from fraction A by HPLC using column B with an isocratic mobile phase (CH_3_CN/H_2_O: 25/75) at a flow rate of 1.0 mL/min. Similarly, by using column B with an isocratic mobile phase (CH_3_CN/H_2_O: 45/55), compound **2** (4.5 mg, *t_R_* 9.32 min) was purified from fraction B, compounds **3** (4.1 mg, *t_R_* 13.54 min) and **4** (1.0 mg, *t_R_* 12.35 min) were obtained from fraction C.

Dioxamycin (**2**): Orange amorphous powder; [α]^23^_D_ +43.8 °C (*c* 0.05, MeOH); UV (*c* 0.05, MeOH) λ_max_ (log ε) 224 (8046), 296 (5172), 425 (652) nm; ^13^C NMR data (125 MHz, in CD_3_OD and DMSO-*d*_6_) and ^1^H NMR data (500 MHz, in CD_3_OD and DMSO-*d*_6_), see Table 1 and Table 2; HRESIMS *m/z* [M + H]^+^ 737.2521 (calcd for C_38_H_41_O_15_, 737.2446), [M + Na]^+^ 759.2330 (calcd for C_38_H_40_NaO_15_, 759.2265).

Fradimycin A (**3**): Orange amorphous powder; [α]^23^_D_ +39.5 °C (*c* 0.05, MeOH); UV (*c* 0.05, MeOH) λ_max_ (log ε) 224 (7578), 296 (5122), 426 (674) nm; ^13^C NMR data (125 MHz, in CD_3_OD and DMSO-*d*_6_) and ^1^H NMR data (500 MHz, in CD_3_OD and DMSO-*d*_6_), see Table 1 and Table 2; HRESIMS *m/z* [M + H]^+^ 751.2673 (calcd for C_39_H_43_O_15_, 751.2602), [M + Na]^+^ 773.2463 (calcd for C_39_H_42_NaO_15_, 773.2421).

Fradimycin B (**4**): Orange amorphous powder; [α]^23^_D_ +36.3 °C (*c* 0.05, MeOH); UV (*c* 0.05, MeOH) λ_max_ (log ε) 222 (7644), 294 (4544), 427 (584) nm; ^13^C NMR data (125 MHz, in CD_3_OD and DMSO-*d*_6_) and ^1^H NMR data (500 MHz, in CD_3_OD and DMSO-*d*_6_), see Table 1 and Table 2; HRESIMS *m/z* [M + H]^+^ 719.2408 (calcd for C_38_H_39_O_14_, 719.2340), [M + Na]^+^ 741.2247 (calcd for C_38_H_38_NaO_14_, 741.2159).

MK844-mF10 (**5**): Orange amorphous powder; [α]^23^_D_ +39.6 °C (*c* 0.01, MeOH); UV (*c* 0.05, MeOH) λ_max_ (log ε) 221 (7612), 297 (4472) , 425 (528) nm; ^13^C NMR data (125 MHz, in DMSO-*d*_6_) and ^1^H NMR data (500 MHz, in DMSO-*d*_6_), see Table 1 and Table 2; HRESIMS *m/z* [M + H]^+^ 703.2380 (calcd for C_38_H_39_O_13_, 703.2391), [M + Na]^+^ 725.2188 (calcd for C_38_H_38_NaO_13_, 725.2210).

Fradic acid A (**6**): colorless powder; [α]^23^_D_ +29.8 °C (*c* 0.01, MeOH); UV (MeOH) λ_max_ 223, 298 nm; ^1^H NMR data (500 MHz, in DMSO-*d*_6_) and ^13^C NMR data (125 MHz, in DMSO-*d*_6_), see Table 3; HRESIMS *m/z* [M − H]^−^ 267.0878 (calcd for C_13_H_1__5_O_6_, 267.0869).

Fradic acid B (**7**): colorless powder; [α]^23^_D_ +29.8 °C (*c* 0.01, MeOH); UV (MeOH) λ_max_ 223, 298 nm; ^1^H NMR data (500 MHz, in DMSO-*d*_6_) and ^13^C NMR data (125 MHz, in DMSO-*d*_6_), see Table 3; HRESIMS *m/z* [M + H]^+^ 283.1177 (calcd for C_1__4_H_1__9_O_6_, 283.1182), [M + Na]^+^ 305.0998 (calcd for C_14_H_18_NaO_6_, 305.1001).

### 3.5. Alkaline Hydrolysis of Compounds **2–4**

Dioxamycin (**2**, 1.0 mg) was refluxed with 2 mL 0.8 M NaOH at 80 °C for 4 h. After cooling, the reaction mixture was neutralized with 1M HCl and then applied to a small column of Diaion HP-20 (30 mL) eluting with 50 mL H_2_O and then 50 mL 80% MeOH. The 80% MeOH elution was evaporated to dryness under reduced pressure. The residue was separated by HPLC to afford compounds **2a** and **6**. By the same method, **3** (0.5 mg) afforded **2a**, and **4** (0.3 mg) afforded **6**.

Compound **2a**: Orange amorphous powder; [α]^23^_D_ +38.6 °C (*c* 0.01, MeOH); UV (MeOH) λ_max_ 220, 296, 429 nm.

### 3.6. Antimicrobial Assay

The previous reported method [5] was used to evaluate the antimicrobial activities of compounds **3–7** against *Staphylococcus aureus* in liquid growth medium. Briefly, a 2.0 mg/mL stock solution of tested compound in DMSO was two-fold serially diluted with DMSO. In a 96-well microtiter plate, 10 μL of each compound solution was mixed with 90 μL of a log-phase growth cells at a dilution of OD_600_ nm = 0.001 in Mueller-Hinton Broth. The final concentrations of test compound were 0.5 to 16.0 μg/mL. After 18 h of incubation at 37 °C, minimal inhibitory concentrations (MIC) were determined by visual inspection.

### 3.7. Tumor Cells Culture

Human colon cancer HCT-15 cells and rat glioma C6 cells were separately cultured in RPMI 1640 and Dulbecco's modified eaglemedium (DMEM) (Gibco) with 10% FBS (PAA) at 37 °C in a humidified, 5% CO_2_ incubator. Human colon cancer SW620 cells were grown in Leibovitz’s L15 medium at 37 °C without CO_2_. Cells after the third generation were used for the experiment.

### 3.8. SRB Assay

Inhibition of cancer cell-growth activity was determined by a Sulforhodamine B (SRB) assay using human colon cancer HCT-15 and SW620 cells as well as rat glioma C6 cells. Briefly, tumor cells were plated in 96-well plate and then treated with different concentrations of tested compound after cells adhesion for 24 h. After 72 h of the treatment, cells were fixed with 50 μL of 10% cold trichloroacetic acid (TCA) solution for 1 h at 4 °C, washed with distilled water five times, and then dried at room temperature. The dried cells were stained with 50 μL of 0.4% SRB for ten minutes and rinsed with 1% acetic acid solution five times. After being dried, dye was dissolved in 10 mM Tris buffer and measured at 515 nm on a microplate reader (Bio-Tech).

### 3.9. Cell Cycle Assay

Cell cycle perturbations induced by the tested compound were analyzed by propidium iodide (PI) DNA staining using flow cytometry. Briefly, human cancer HCT-15 cells were treated with the tested compound for 3, 6, 12 and 24 h. At the end of the treatment, cells were harvested and prepared as a single cell in icy phosphate buffered saline (PBS), and then the cells were fixed with ice-cold 70% ethanol at 4 °C overnight. Cells were harvested by centrifugation (1900 rpm, 7 min) and washed with PBS twice, resuspended in PBS with RNase A (50 U/mL), then incubated for 30 min at 37 °C, and finally stained with PI in the dark at 4 °C for 30 min. Cell cycle distribution were studied using a FACScan flow cytometry.

### 3.10. Morphological Analysis of Apoptosis

Hoechst 33342 and propidium iodide (PI) double staining were used for detecting cell apoptosis and necrosis. Tumor cells were incubated with the tested compound at different concentrations in an atmosphere with 5% CO_2_ at 37 °C for 72 h, and then cells were stained with 10 μg/mL Hoechst 33342 and 5 μg/mL PI for 20 min at room temperature. After being washed with PBS twice, fluorescence was imaged by fluorescence microscope at 40× magnification. Apoptotic cells stained by Hoechst 33342 showed bright blue nuclear condensation and fragmentation, and necrotic cells displayed red fluorescence as stained by PI.

### 3.11. Annexin V-FITC/PI Double Staining Assay

Apoptosis and necrosis were quantified using an Annexin V apoptosis detection kit. Cells were treated with the tested compound for 24 h and 72 h, and then 1 × 10^6^ cells were harvested. The cells were washed in cold PBS buffer and further resuspended in 100 μL 1× binding buffer mixed with 5 μL Annexin V-FITC and 1 μL 100 μg/mL PI working solution. Cells were incubated at room temperature for 15 min and then 400 μL 1× binding buffer was added. Fluorescence was analyzed by flow cytometry at the fluorescence emission at 530 nm and 575 nm using 488 nm excitation.

## 4. Conclusions

Four new compounds of fradimycins A (**3**) and B (**4**) and fradic acids A (**6**) and B (**7**) were isolated and identified from marine *Streptomyces fradiae* PTZ0025. Fradimycins A (**3**) and B (**4**) showed potent antibacterial activity against *Staphylococcus aureus* and significantly inhibited cell-growth of human colon cancer HCT-15 and SW620 cells as well as rat glioma C6 cells. Fradimycin B (**4**) induced apoptosis and necrosis of HCT-15, SW620, C6 cells and blocked HCT-15 cells at the G_0_/G_1_ phase. Taken together, the results from this study demonstrated that the new marine natural products fradimycins A (**3**) and B (**4**), particularly fradimycin B (**4**) from marine *Streptomyces fradiae* strain PTZ0025 had potent antimicrobial and antitumor activities.

## Supplementary Files

Supplementary File 1: PDF-Document (PDF, 768 KB)

## References

[1] Hayakawa Y., Iwakiri T., Imamura K., Seto H., Otake N. (1985). Studies on the isotetracenone antibiotics. I. Capoamycin, a new antitumor antibiotic. J. Antibiot..

[2] Hayakawa Y., Adachi K., Iwakiri T., Imamura K., Furihata K., Seto H., Otake N. (1987). Capoamycin, a new isotetracenone antibiotic. Agric. Biol. Chem..

[3] Sawa R., Matsuda N., Uchida T., Ikeda T., Sawa T., Naganawa H., Hamada M., Takeuchi T. (1991). Dioxamycin, a new benz[α]anthraquinone antibiotic. J. Antibiot..

[4] Igarashi M., Utsumi R., Watanabe T. (2011). Novel antibacterial anthraquinone compound, MK844-mF10, microbial manufacture thereof, *Streptomyces* producing it, and compositions containing it. Japan Patent.

[5] Peoples A.J., Zhang Q., Millett W.P., Rothfeder M.T., Pescatore B.C., Madden A.A., Ling L.L., Moore C.M. (2008). Neocitreamicins I and II, novel antibiotics with activity against methicillin-resistant *Staphylococcus aureus* and vancomycin-resistant *Enterococci*. J. Antibiot..

