# Supplementary material for: New Capoamycin-Type Antibiotics and Polyene Acids from Marine Streptomyces fradiae PTZ0025
Source: Mar Drugs. 2012 Oct 29;10(11):2388–402. doi: 10.3390/md10112388 (PMC3509524; doi:10.3390/md10112388)

Supplementary Information

Figure S1. ^{1}H-NMR spectrum of **2** (in CD_3OD). 3
Figure S2. ^{1}H-NMR spectrum of **2** (in DMSO-d_6). 3
Figure S3. ^{13}C-NMR spectrum of **2** (in CD_3OD). 4
Figure S4. ^{13}C-NMR spectrum of **2** (in DMSO-d_6). 4
Figure S5. HMQC spectrum of **2** (in CD_3OD). 5
Figure S6. HMQC spectrum of **2** (in DMSO-d_6). 5
Figure S7. HMBC spectrum of **2** (in DMSO-d_6). 6
Figure S8. UV spectrum of **2**. 6
Figure S9. HRESIMS spectrum of **2**. 7
Figure S10. ^{1}H-NMR spectrum of **3** (in CD_3OD). 8
Figure S11. ^{1}H-NMR spectrum of **3** (in DMSO-d_6). 9
Figure S12. ^{13}C-NMR spectrum of **3** (in CD_3OD). 10
Figure S13. ^{13}C-NMR spectrum of **3** (in DMSO-d_6). 10
Figure S14. HMQC spectrum of **3** (in CD_3OD). 11
Figure S15. HMQC spectrum of **3** (in DMSO-d_6). 11
Figure S16. HMBC spectrum of **3** (in CD_3OD). 12
Figure S17. ROESY spectrum of **3** (in DMSO-d_6). 12
Figure S18. HRESIMS spectrum of **3**. 13
Figure S19. UV spectrum of **3**. 14
Figure S20. ^{1}H-NMR spectrum of **4** (in CD_3OD). 15
Figure S21. ^{1}H-NMR spectrum of **4** (in DMSO-d_6). 16
Figure S22. ^{13}C-NMR spectrum of **4** (in CD_3OD). 17
Figure S23. ^{13}C-NMR spectrum of **4** (in DMSO-d_6). 17
Figure S24. HMQC spectrum of **4** (in CD_3OD). 18
Figure S25. HMBC spectrum of **4** (in CD_3OD). 18
Figure S26. COSY spectrum of **4** (in CD_3OD). 19
Figure S27. UV spectrum of **4**. 19
Figure S28. HRESI MS spectrum of **4**. 20
Figure S29. ^{1}H-NMR spectrum of **5** (in DMSO-d_6). 21
Figure S30. ^{13}C-NMR spectrum of **5** (in DMSO-d_6). 21
Figure S31. HMQC spectrum of **5** (in DMSO-d_6). 22
Figure S32. HRESIMS spectrum of **5** (in DMSO-d_6). 23
Figure S33. UV spectrum of **5**. 24
Figure S34. ^{1}H-NMR spectrum of **6** (in DMSO-d_6). 25
Figure S35. ^{13}C-NMR spectrum of **6** (in DMSO-d_6). 26
Figure S36. HMQC spectrum of **6** (in DMSO-d_6). 26
Figure S37. HMBC spectrum of **6** (in DMSO-d_6). 27
Figure S38. HRESI MS spectrum of **6** (in DMSO-d_6). 27
Figure S39. ^{1}H-NMR spectrum of **7** (in DMSO-d_6). 28
Figure S40. ^{13}C-NMR spectrum of **7** (in DMSO-d_6). 29
Figure S41. HMQC spectrum of **7** (in DMSO-d_6). 29

Figure S42. HRESI MS spectrum of **7**. 30
Figure S43. HPLC profiles of the ethanol extract and the active 90% MeOH fraction. 30
Figure S44. 16S rDNA sequence data of marine *Streptomyces fradiae* strain PTZ0025. 31
Figure S45. Fradimycin B (**4**) induced apoptosis and necrosis in SW620 cells. 32
Figure S46. Fradimycin B (**4**) induced apoptosis and necrosis in C_6 cells. 33

Figure S1. ^{1}H-NMR spectrum of **2** (in CD_3OD).

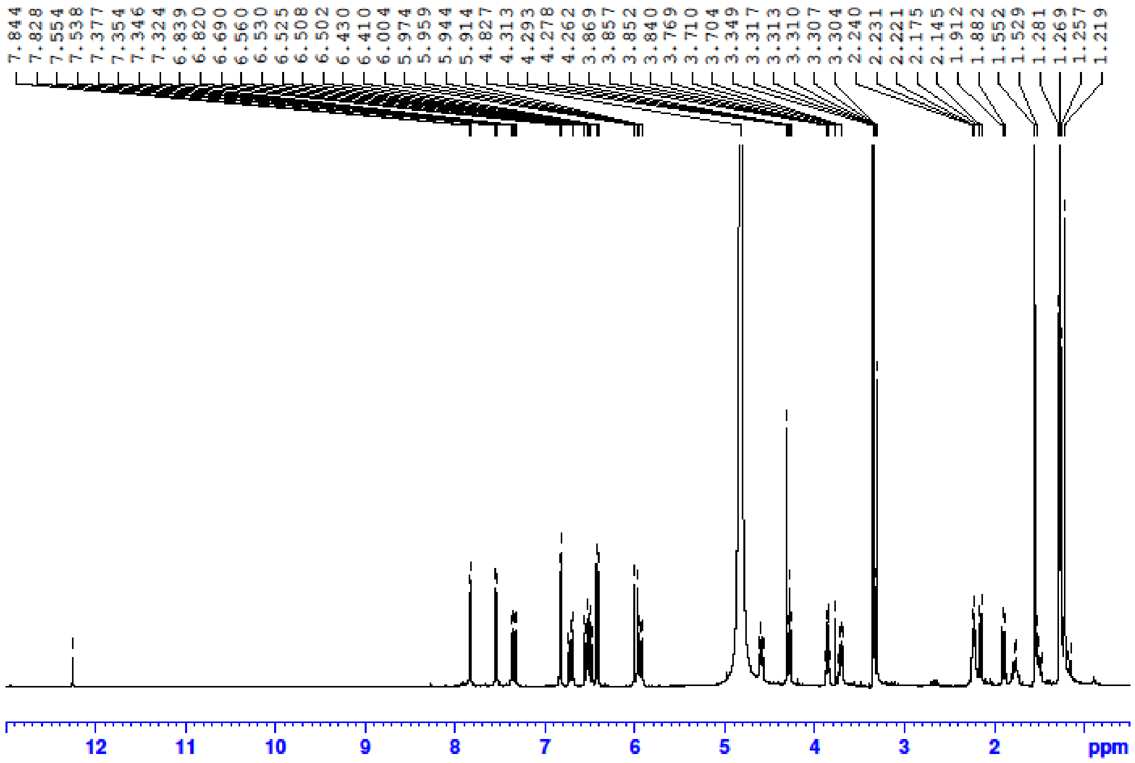

Figure S2. ^{1}H-NMR spectrum of **2** (in DMSO-d_6).

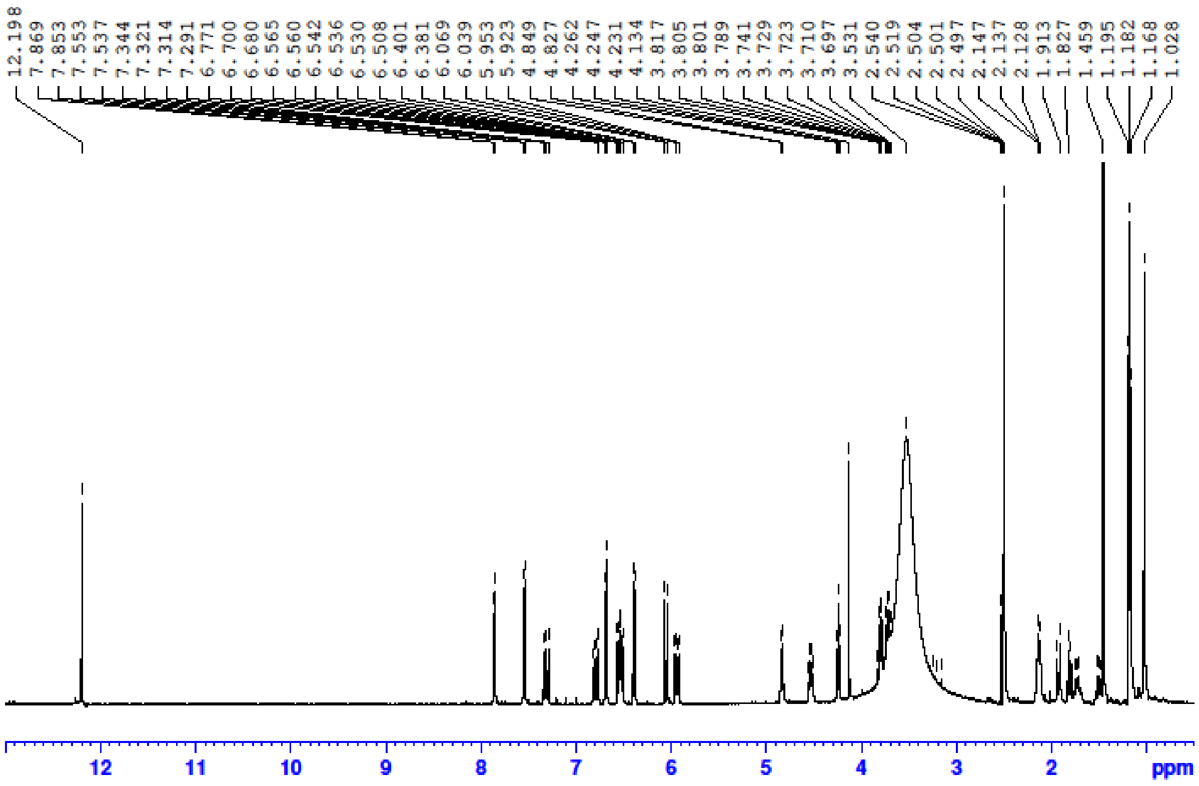

Figure S3. ^{13}C-NMR spectrum of **2** (in CD_3OD).

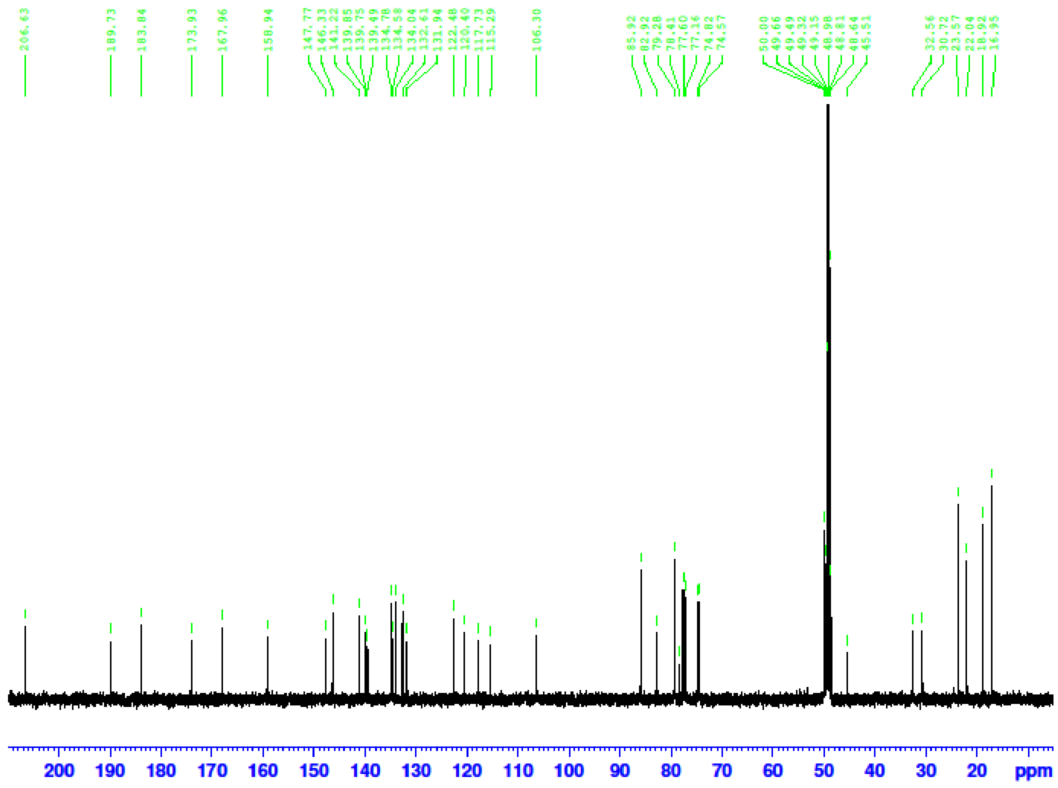

Figure S4. ^{13}C-NMR spectrum of **2** (in DMSO-d_6).

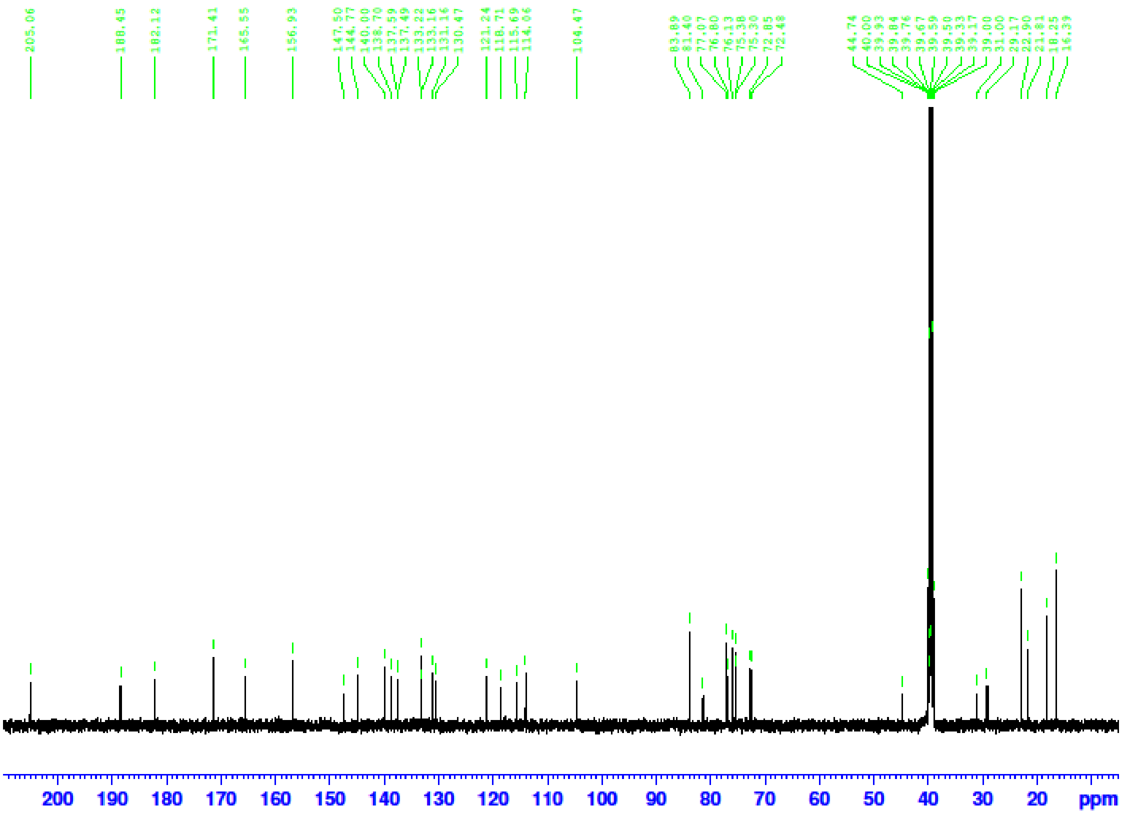

Figure S5. HMQC spectrum of **2** (in CD_3OD).

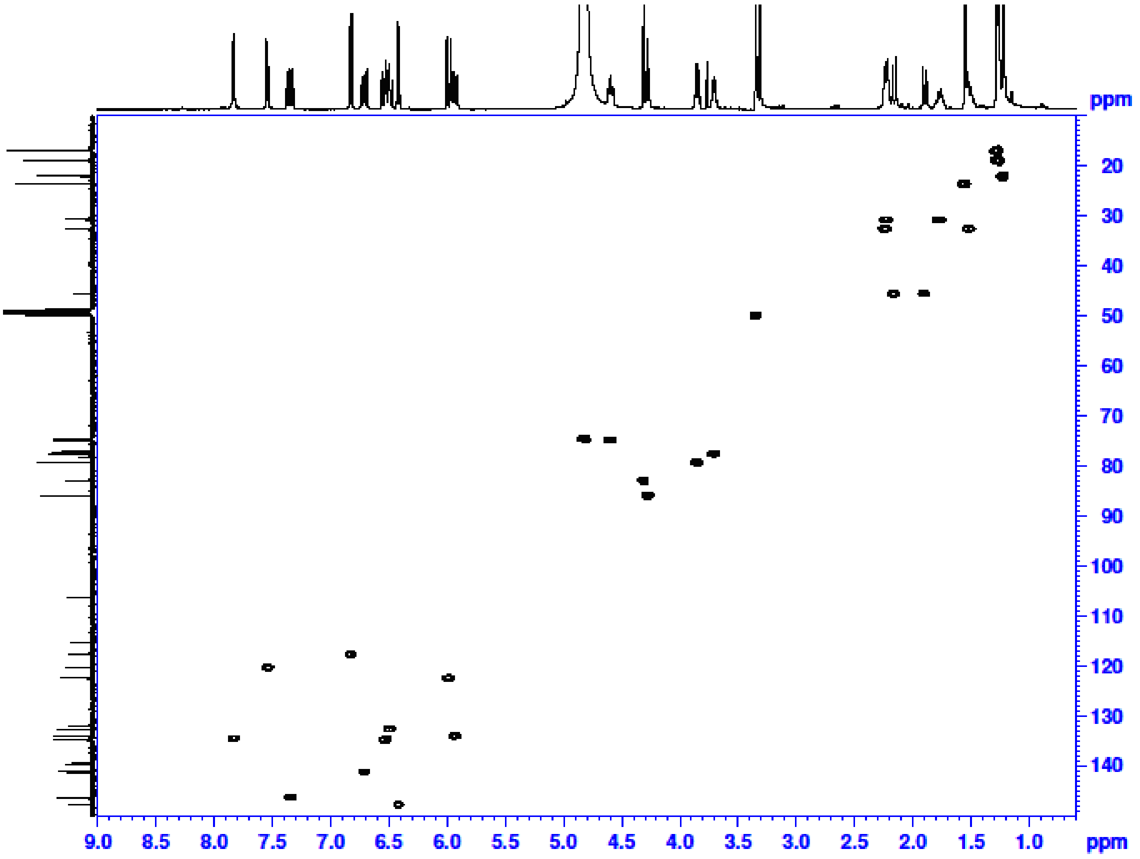

Figure S6. HMQC spectrum of **2** (in DMSO-d_6).

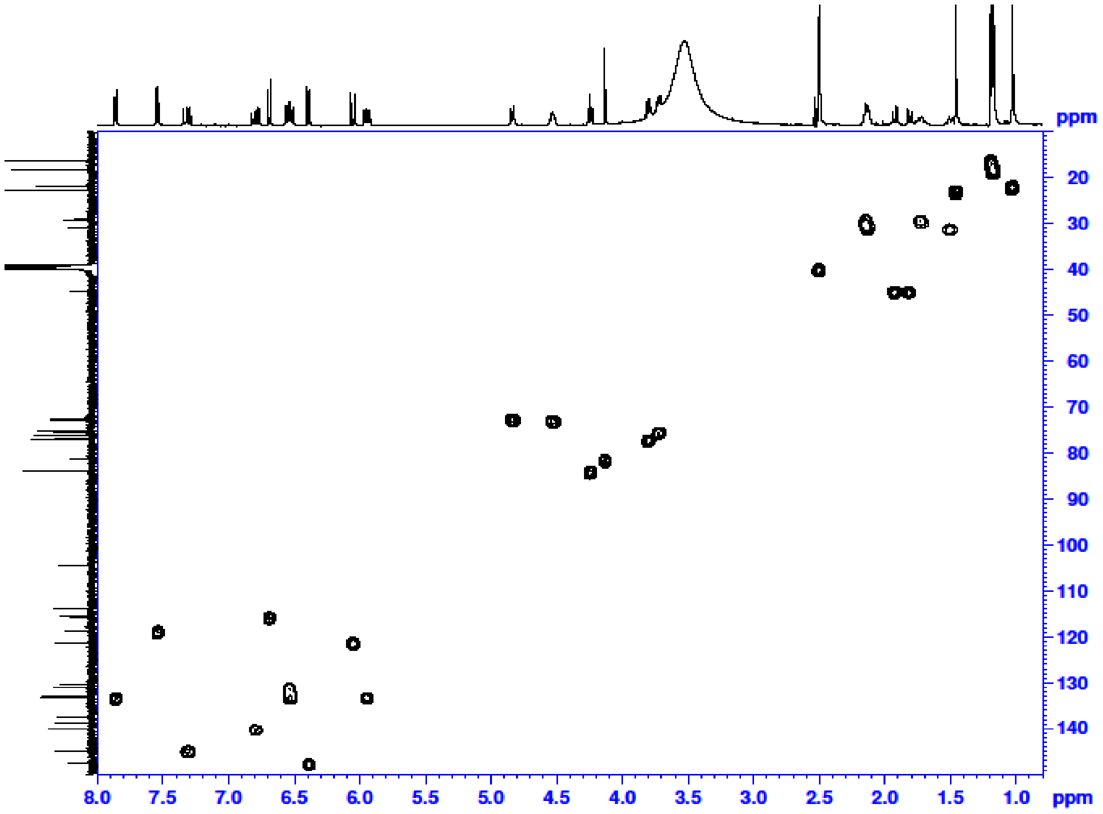

Figure S7. HMBC spectrum of **2** (in DMSO-d_6).

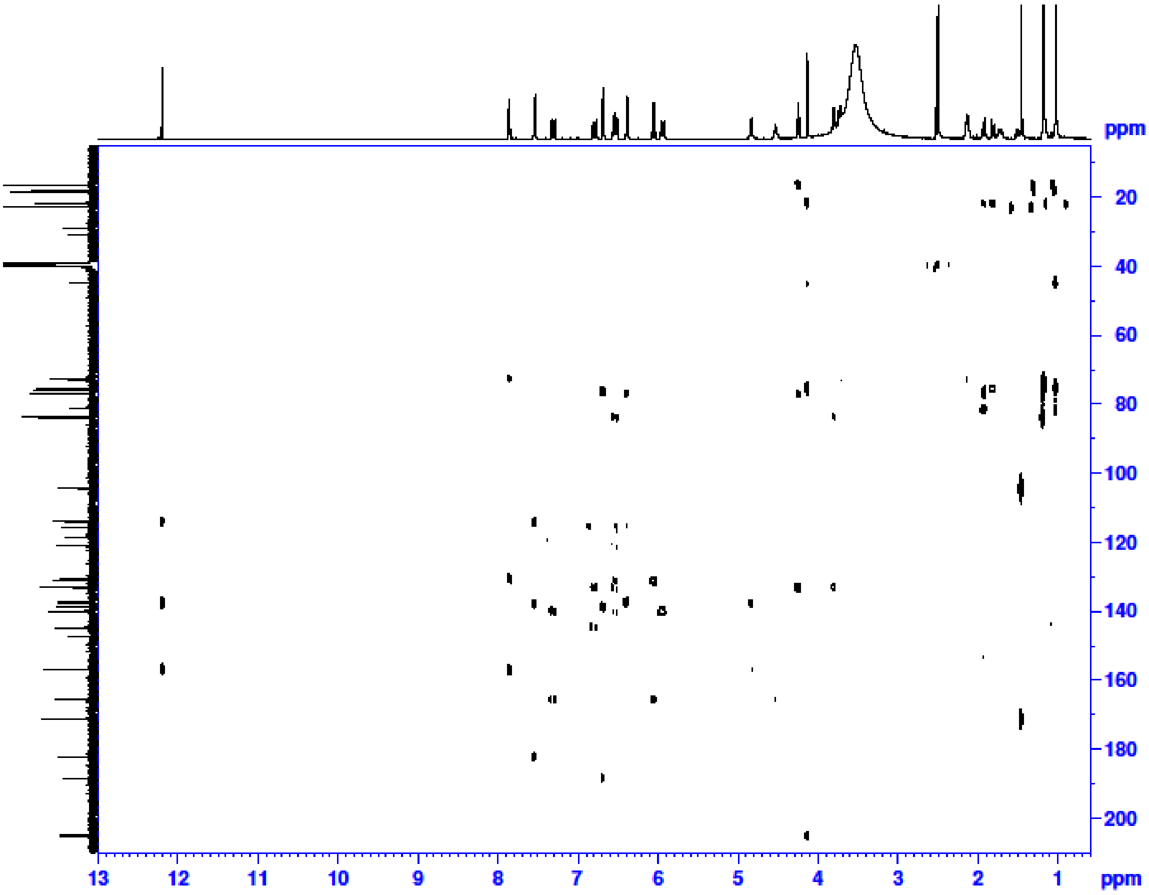

Figure S8. UV spectrum of **2**.

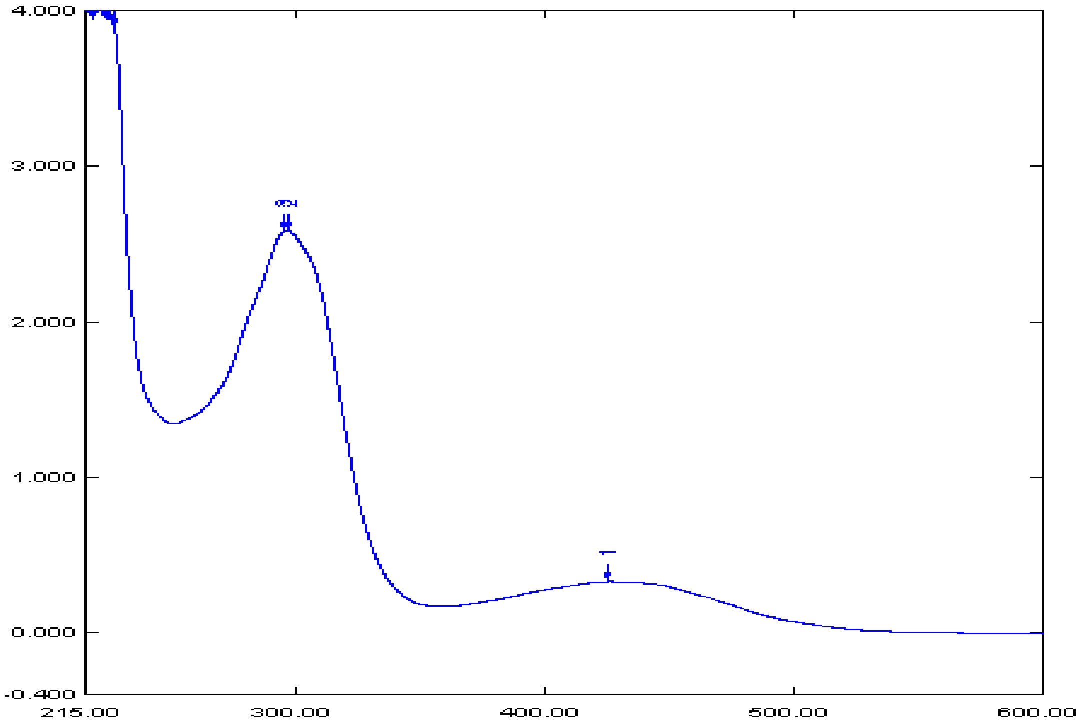

Figure S9. HRESIMS spectrum of **2**.

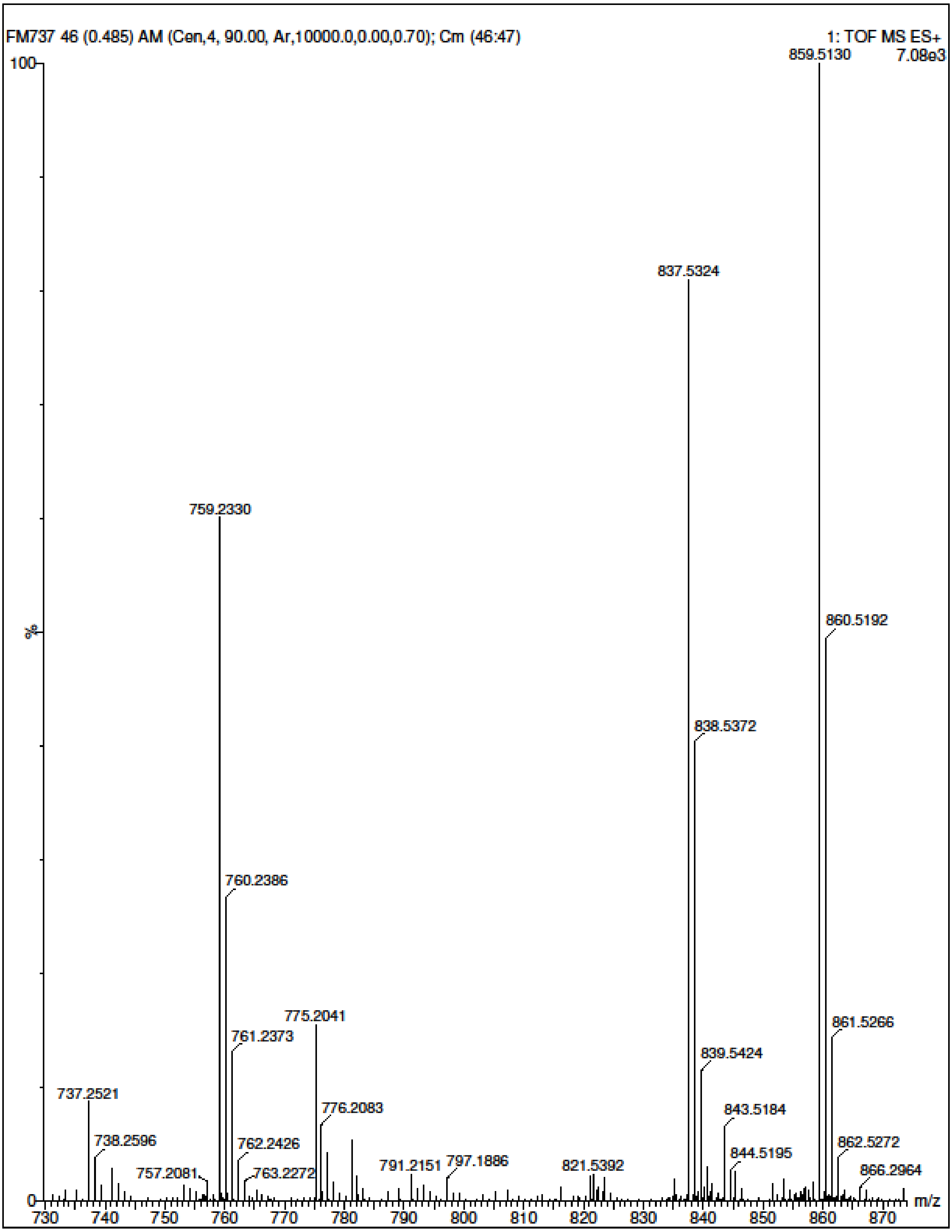

Figure S10. ^{1}H-NMR spectrum of **3** (in CD_3OD).

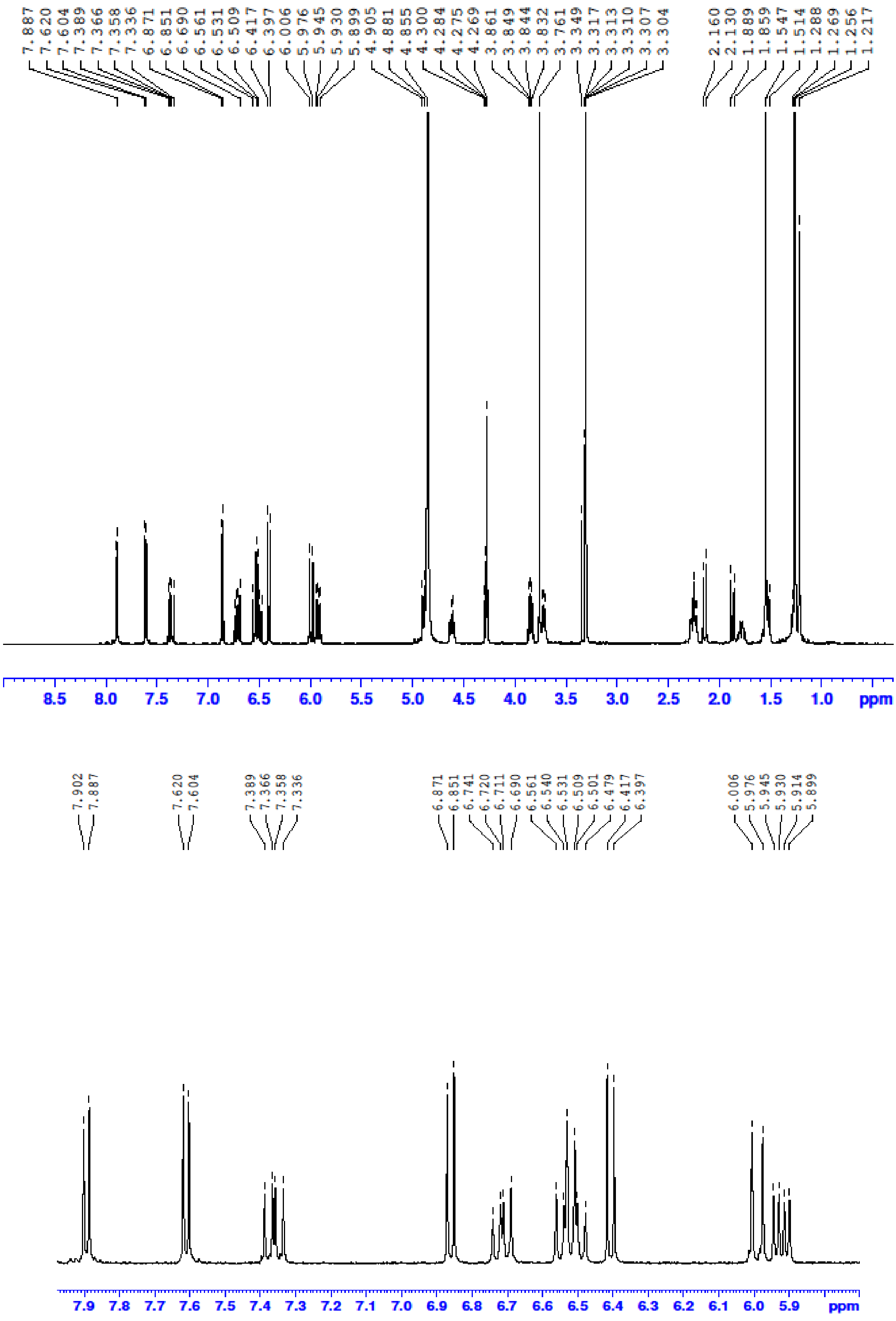

Figure S11. ^{1}H-NMR spectrum of **3** (in DMSO-d_6).

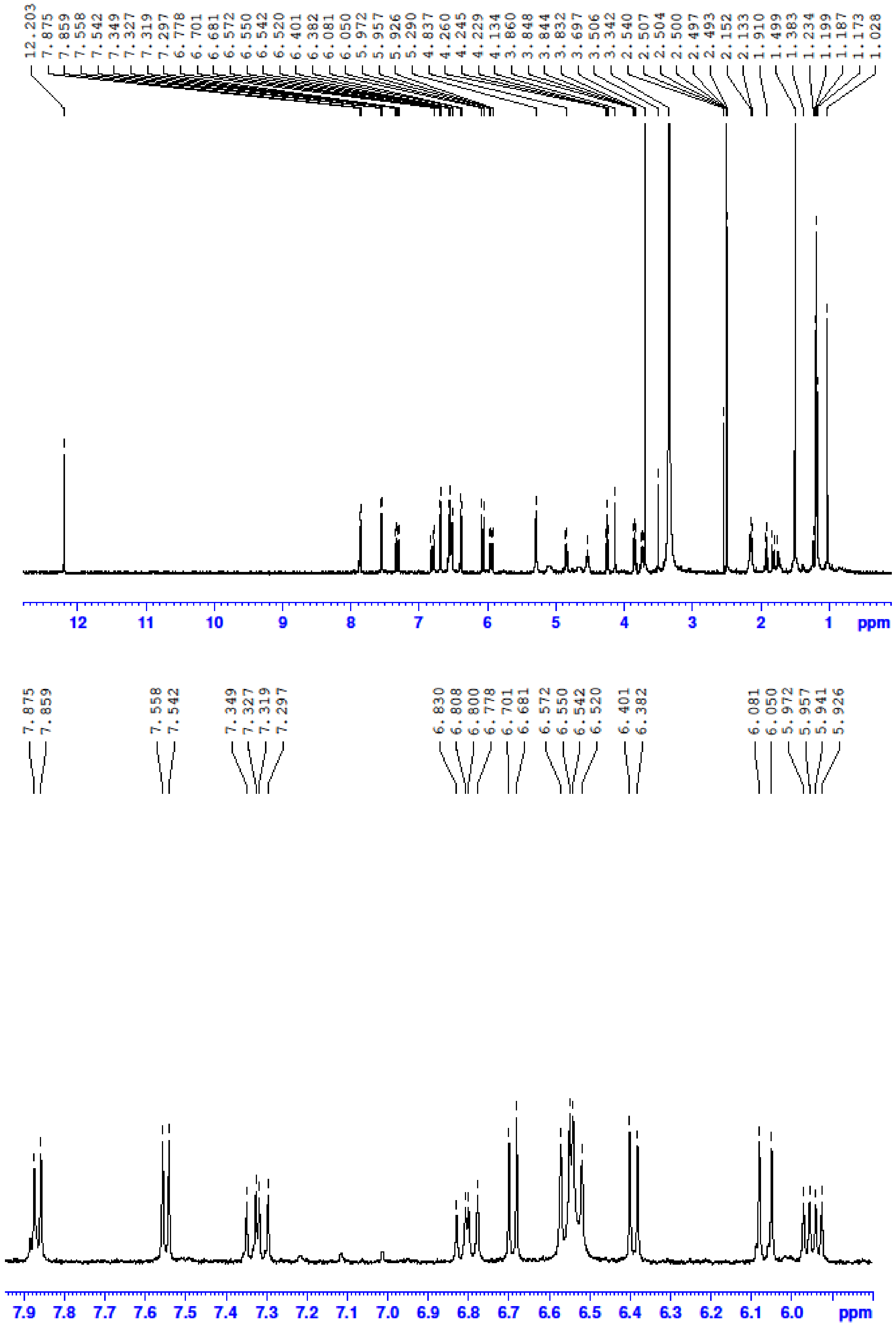

Figure S12. ^{13}C-NMR spectrum of **3** (in CD_3OD).

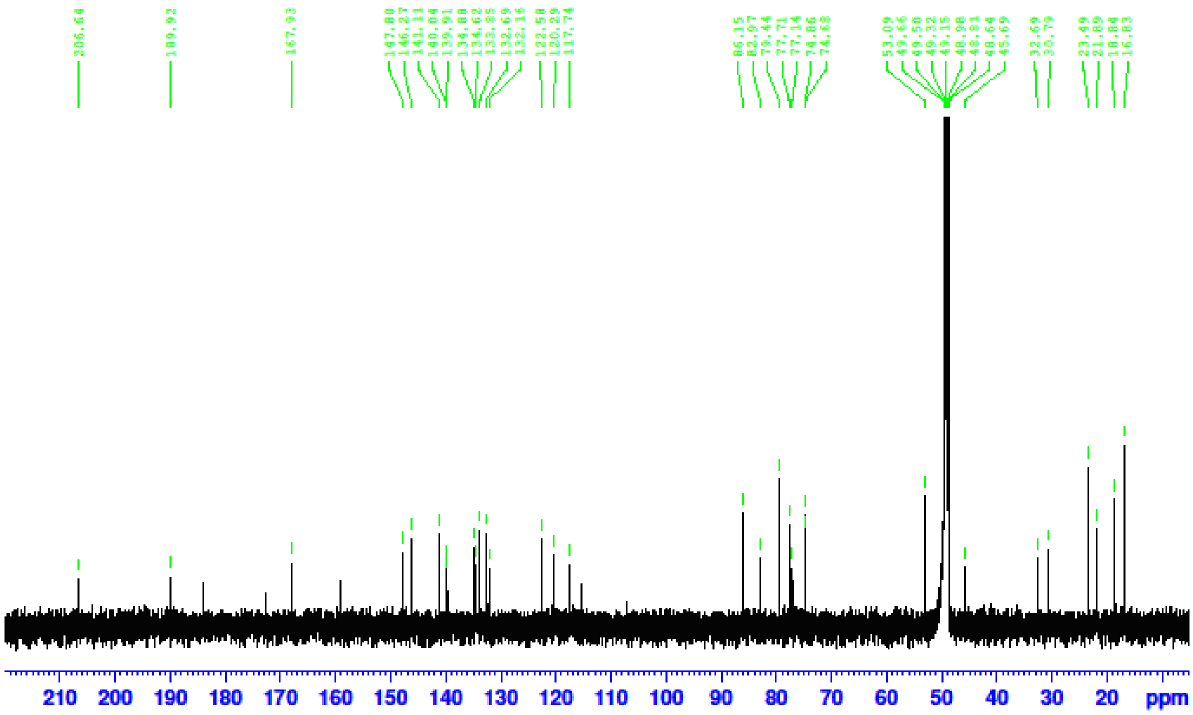

Figure S13. ^{13}C-NMR spectrum of **3** (in DMSO-d_6).

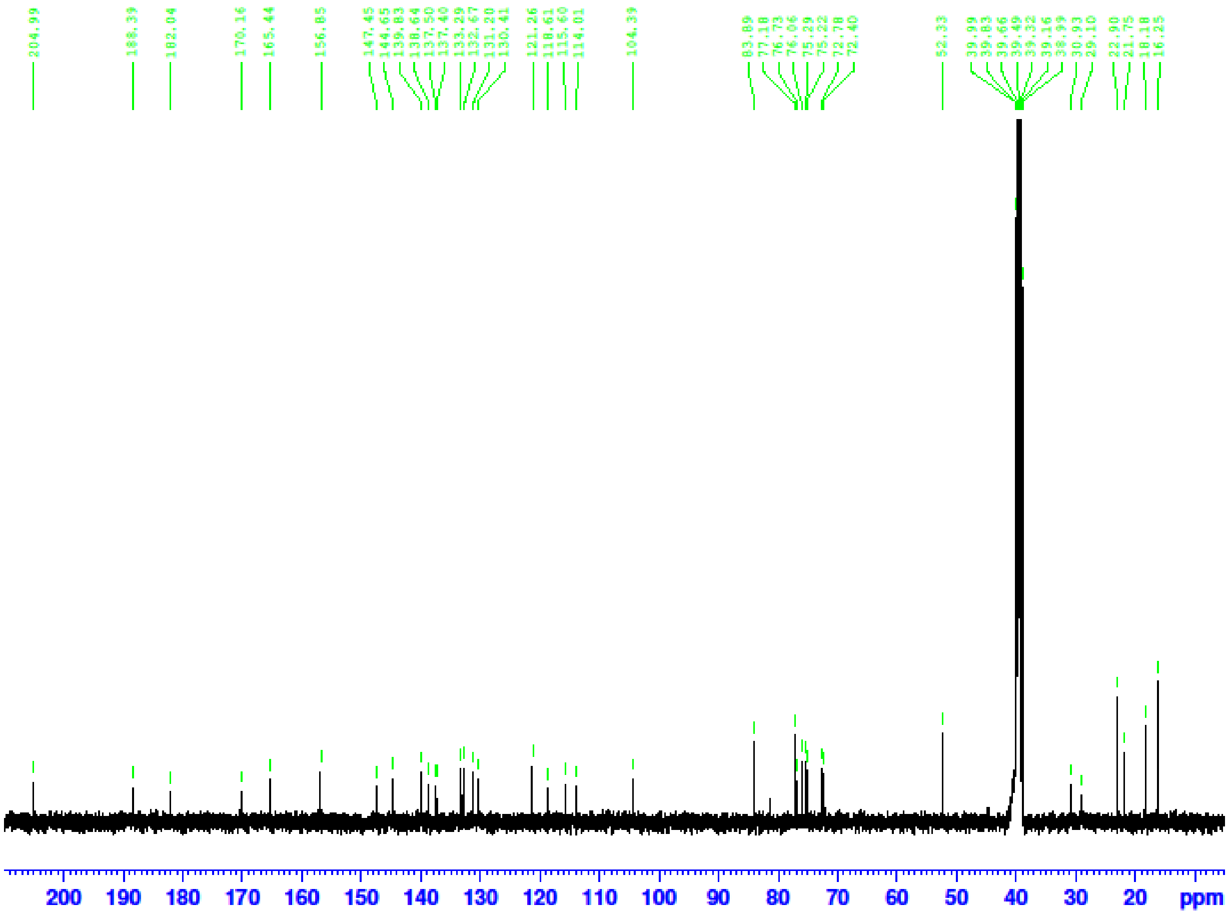

Figure S14. HMQC spectrum of **3** (in CD_3OD).

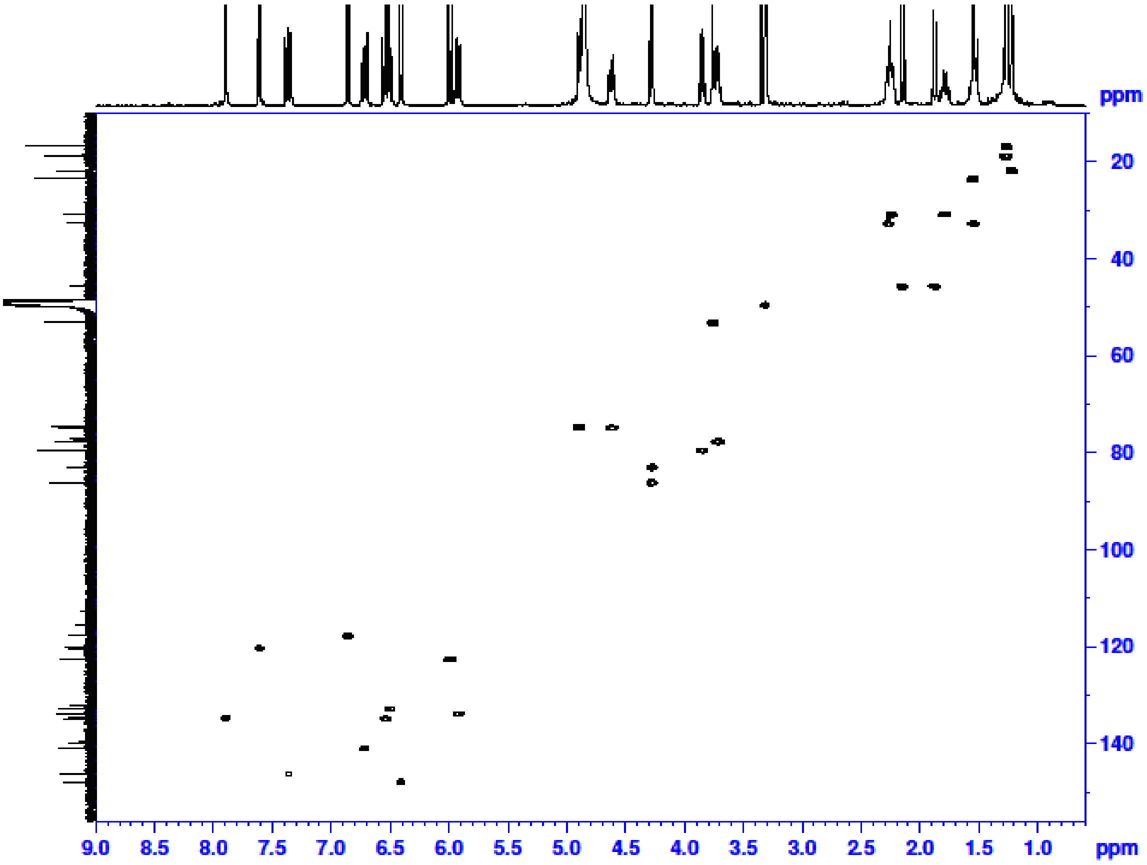

Figure S15. HMQC spectrum of **3** (in DMSO-d_6).

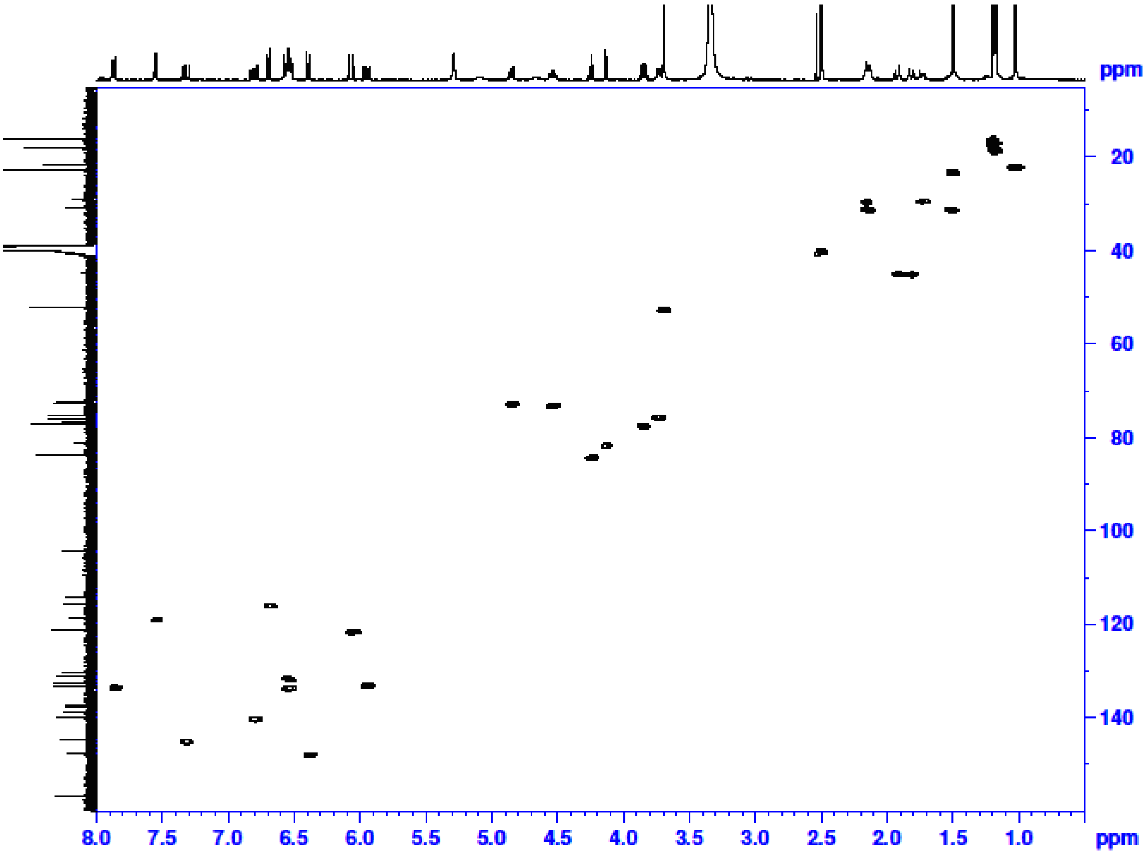

Figure S16. HMBC spectrum of **3** (in CD_3OD).

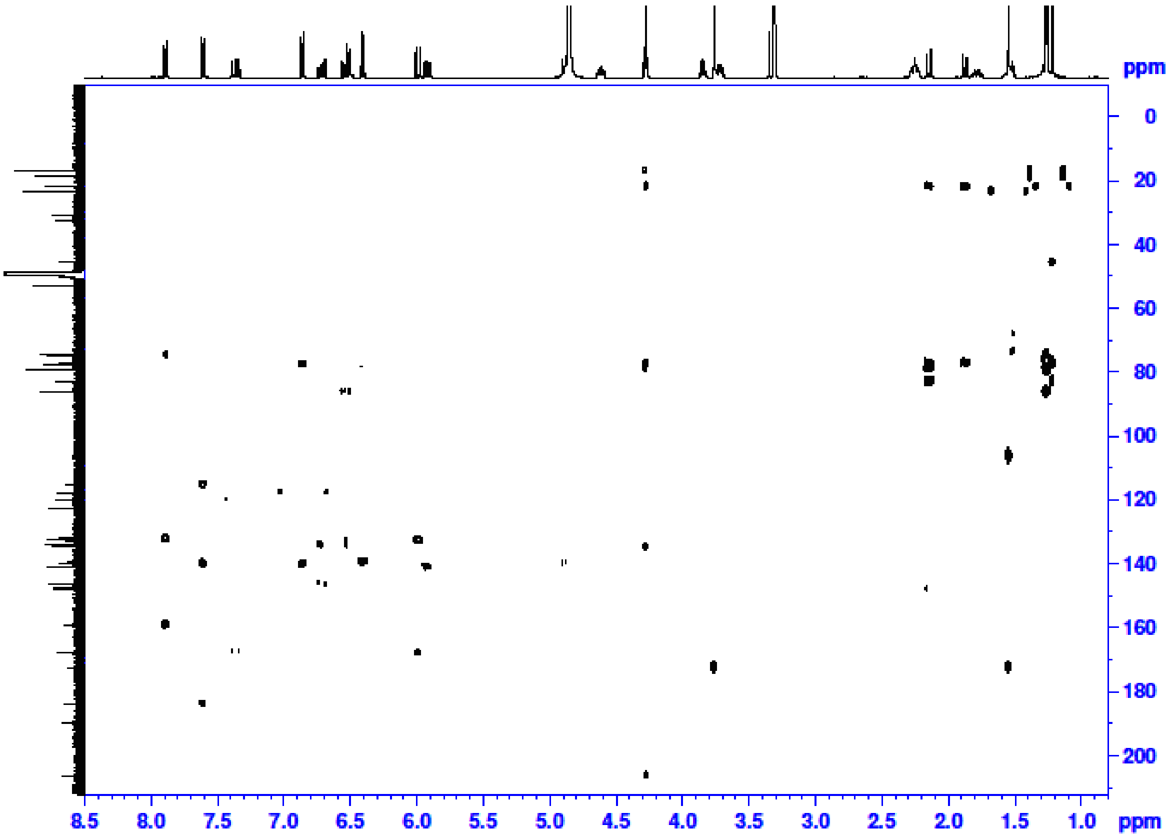

Figure S17. ROESY spectrum of **3** (in DMSO-d_6).

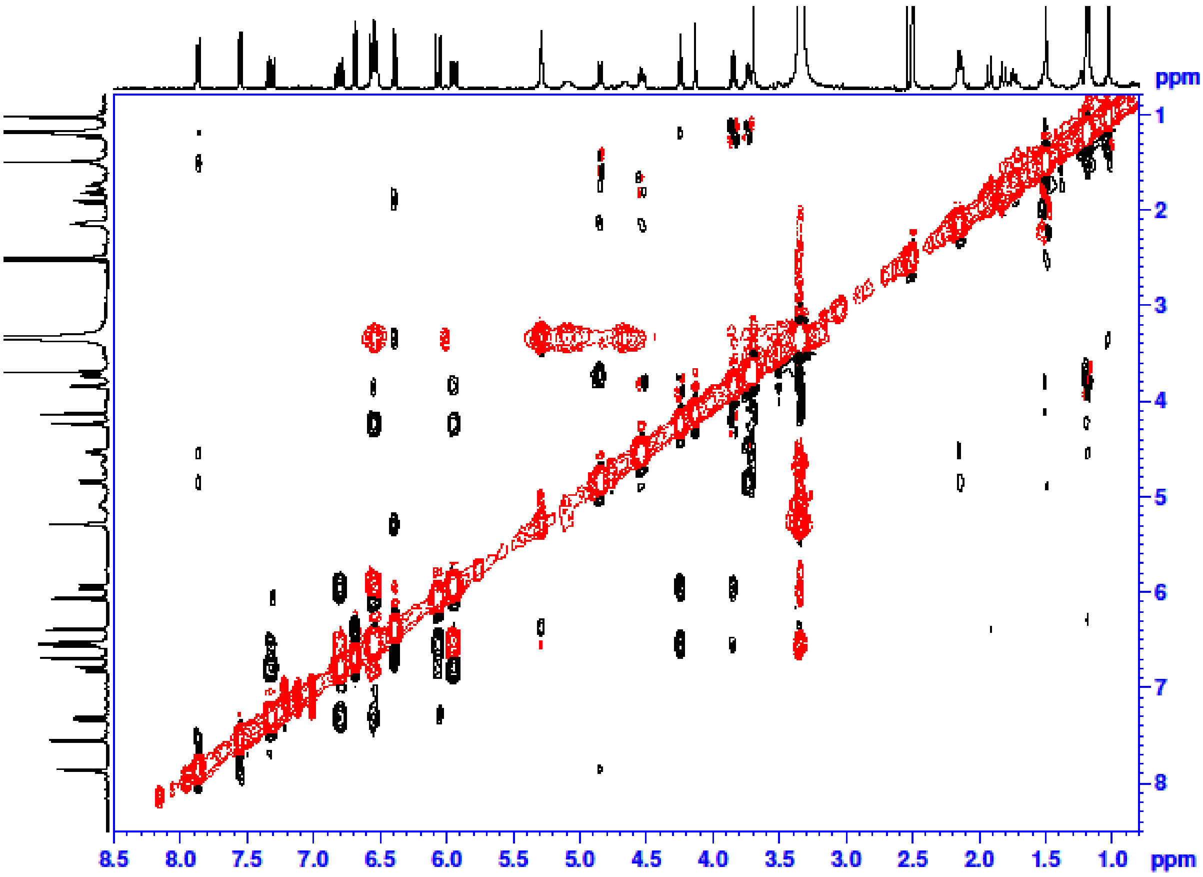

Figure S18. HRESIMS spectrum of **3**.

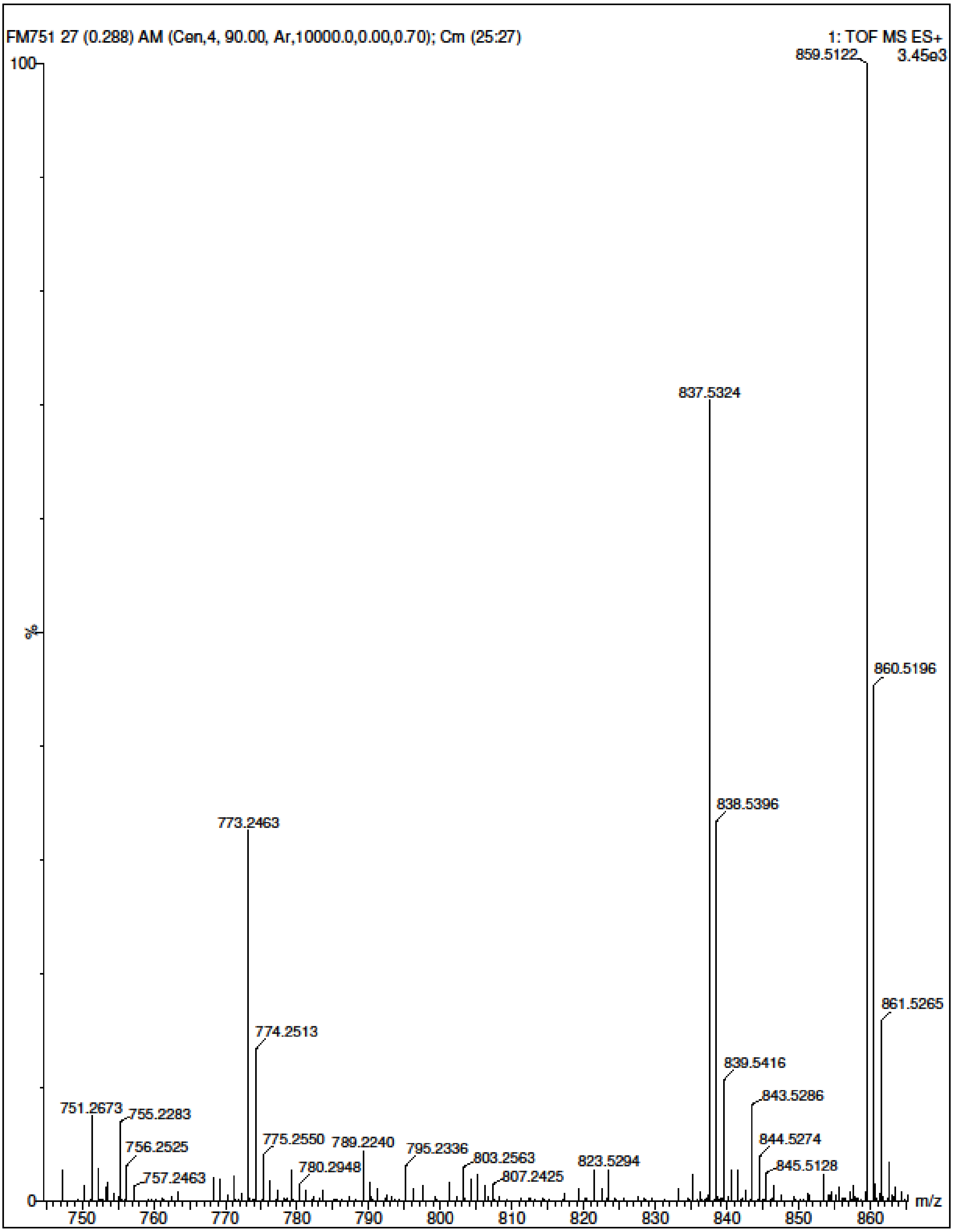

Figure S19. UV spectrum of **3**.

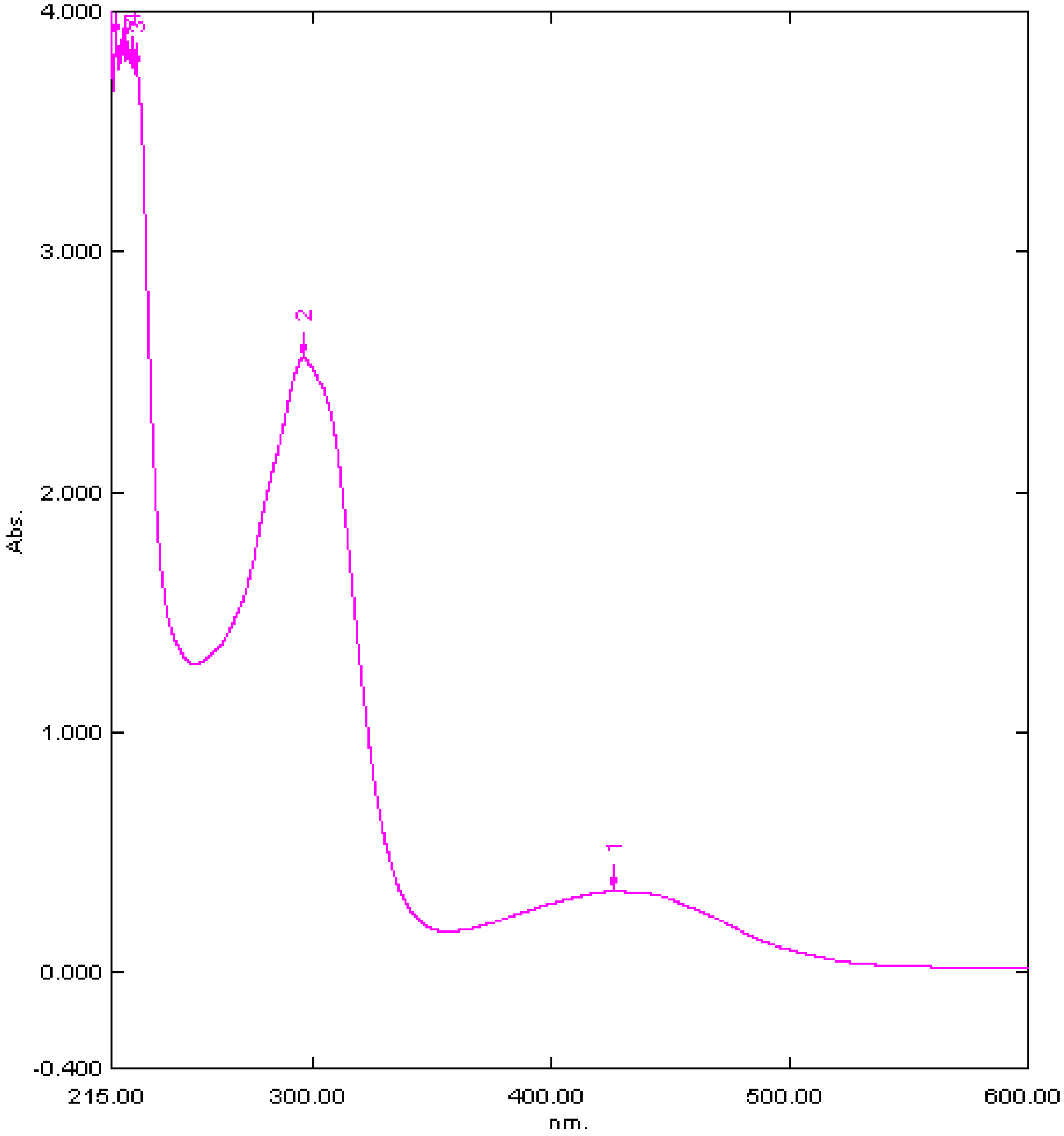

Figure S20. ^{1}H-NMR spectrum of **4** (in CD_3OD).

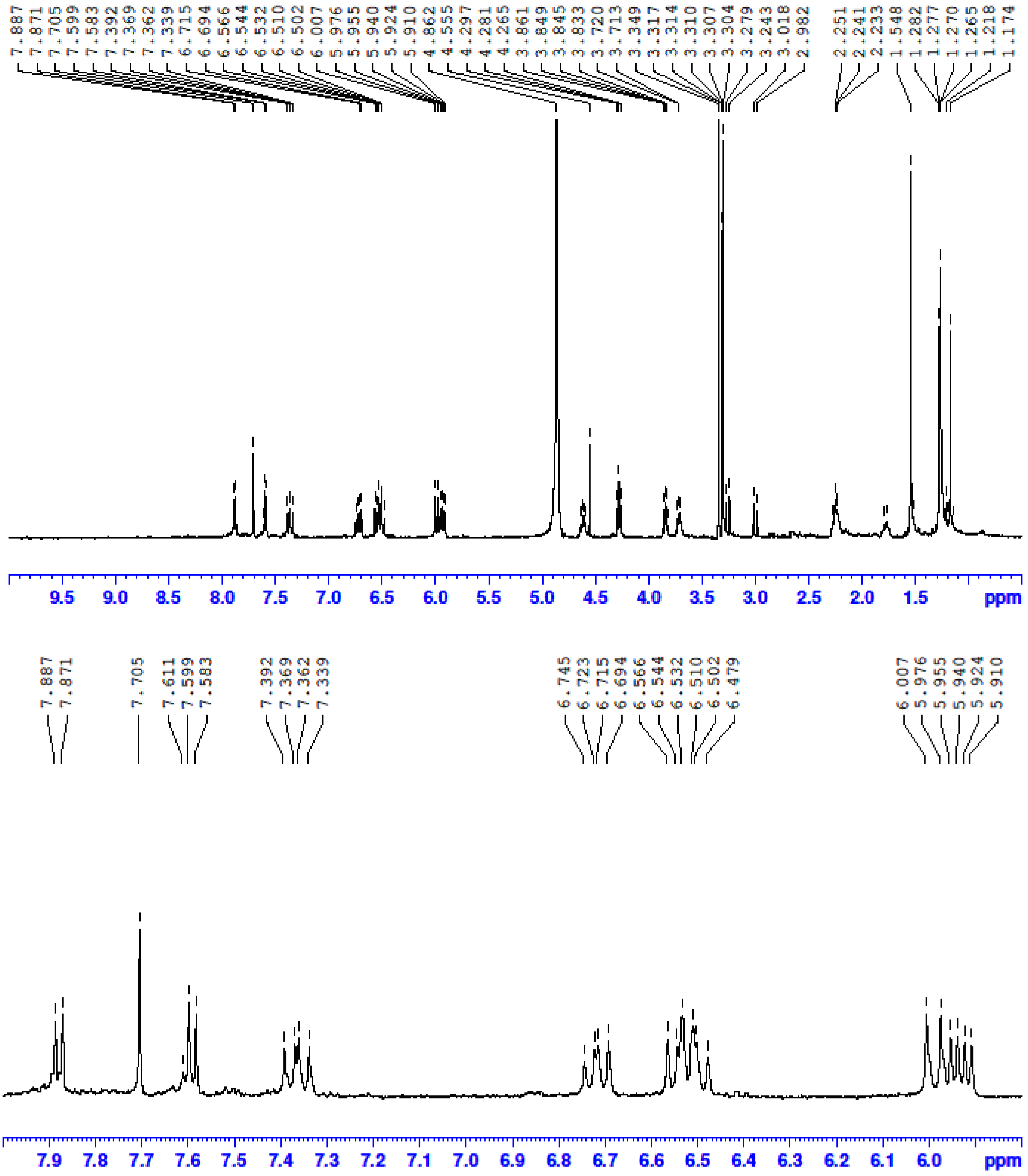

Figure S21. ^{1}H-NMR spectrum of **4** (DMSO-d_6).

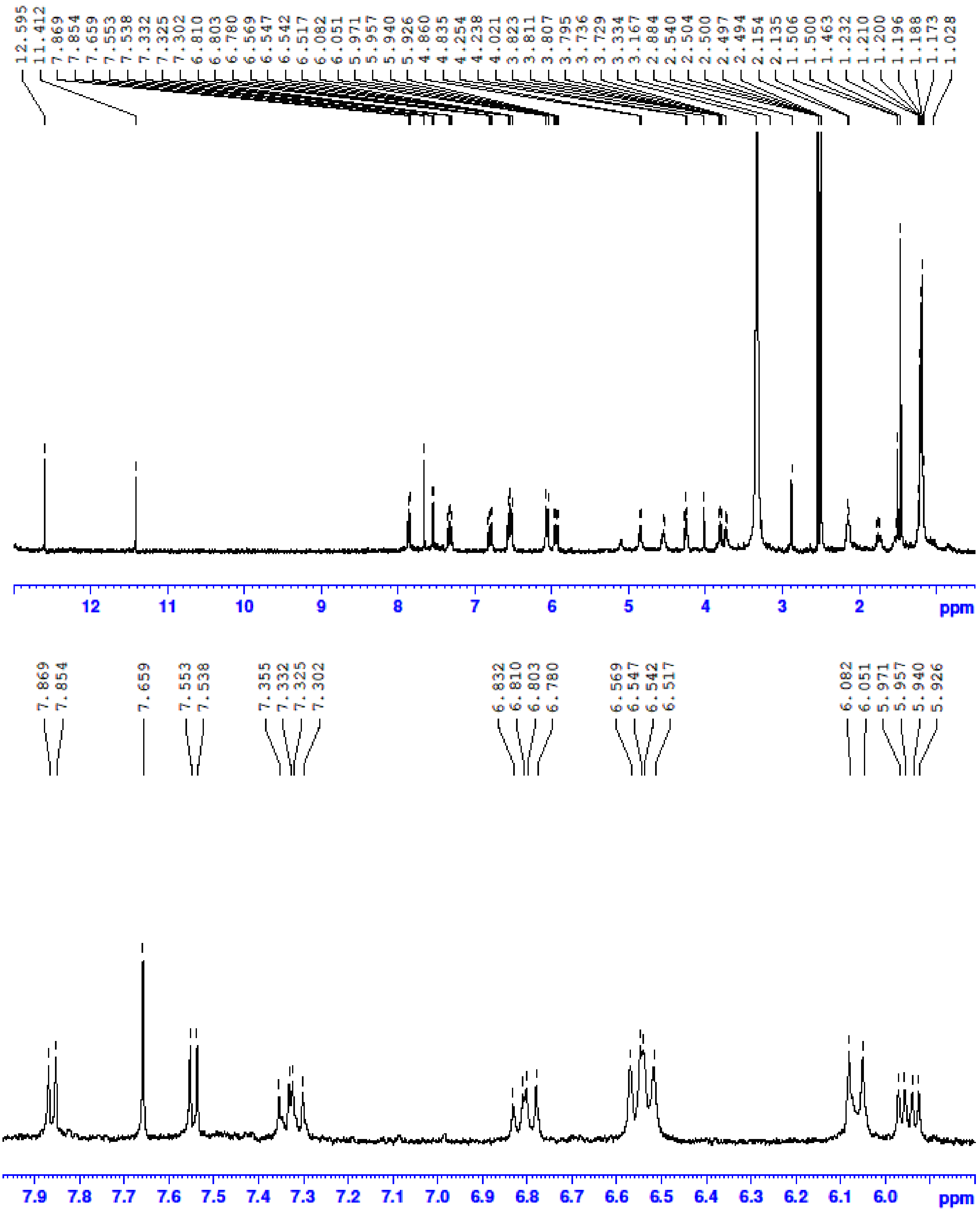

Figure S22. ^{13}C-NMR spectrum of **4** (in CD_3OD).

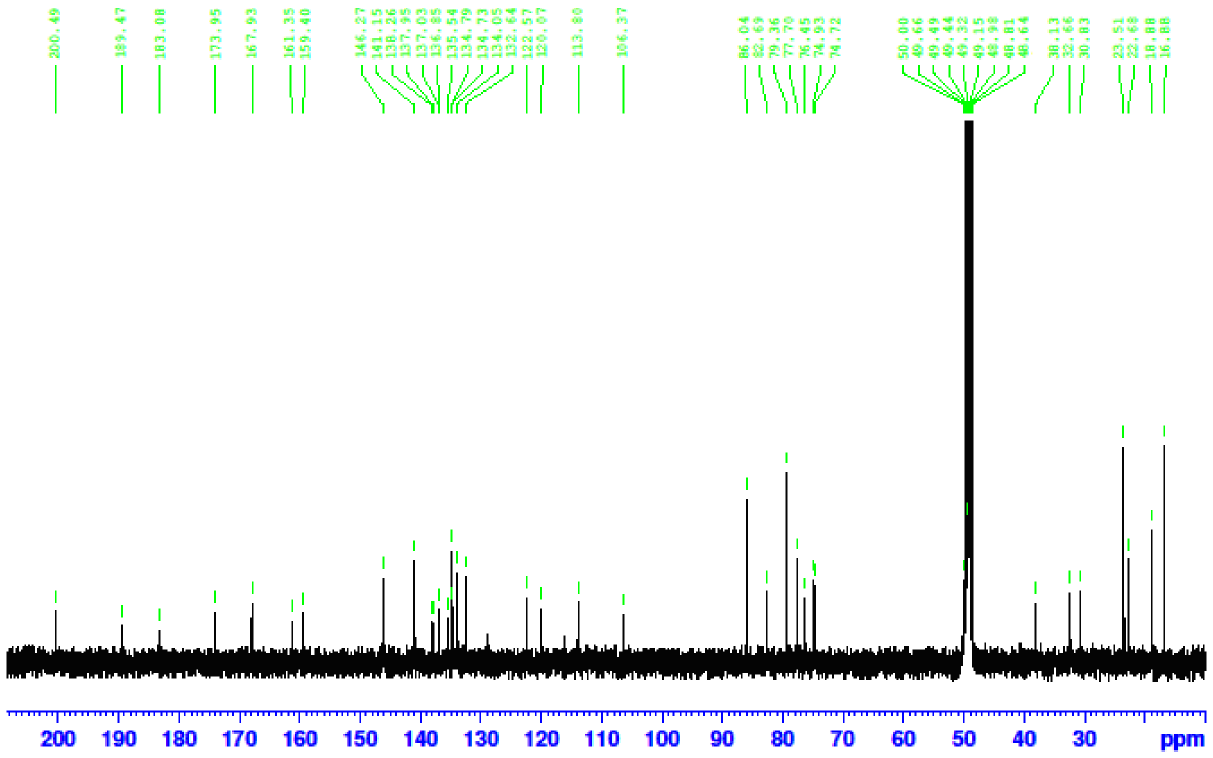

Figure S23. ^{13}C-NMR spectrum of **4** (in DMSO-d_6).

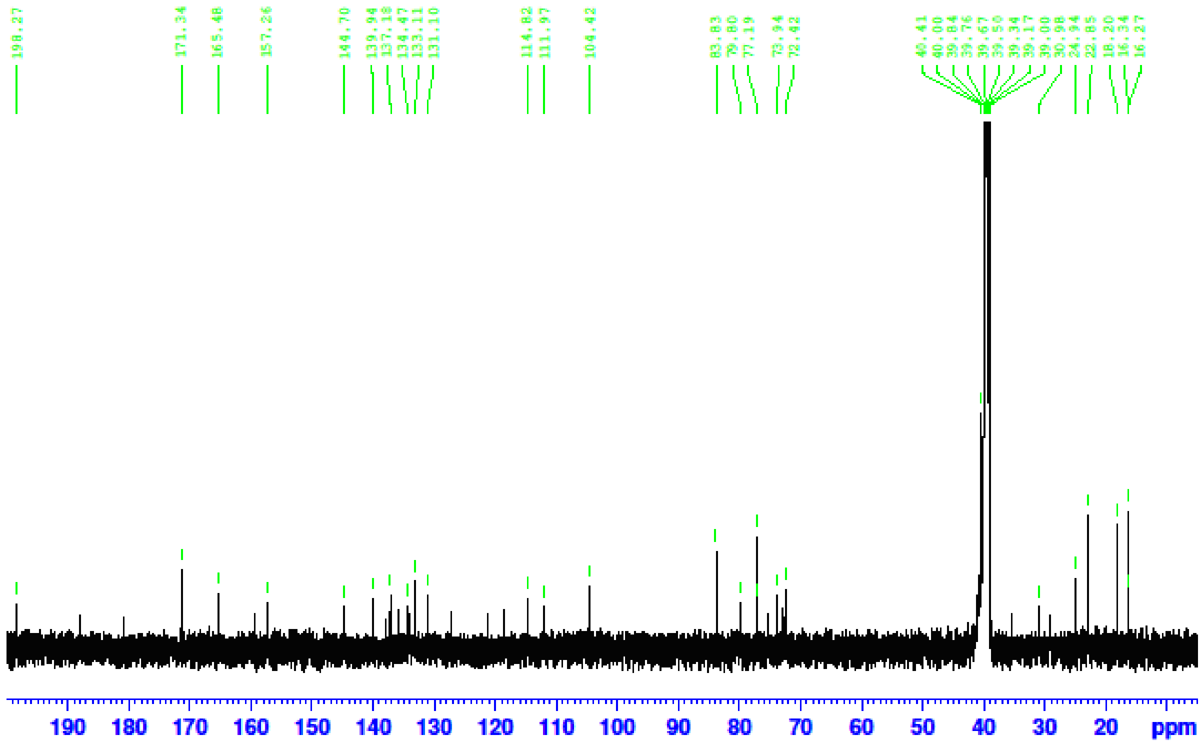

Figure S24. HMQC spectrum of **4** (in CD_3OD).

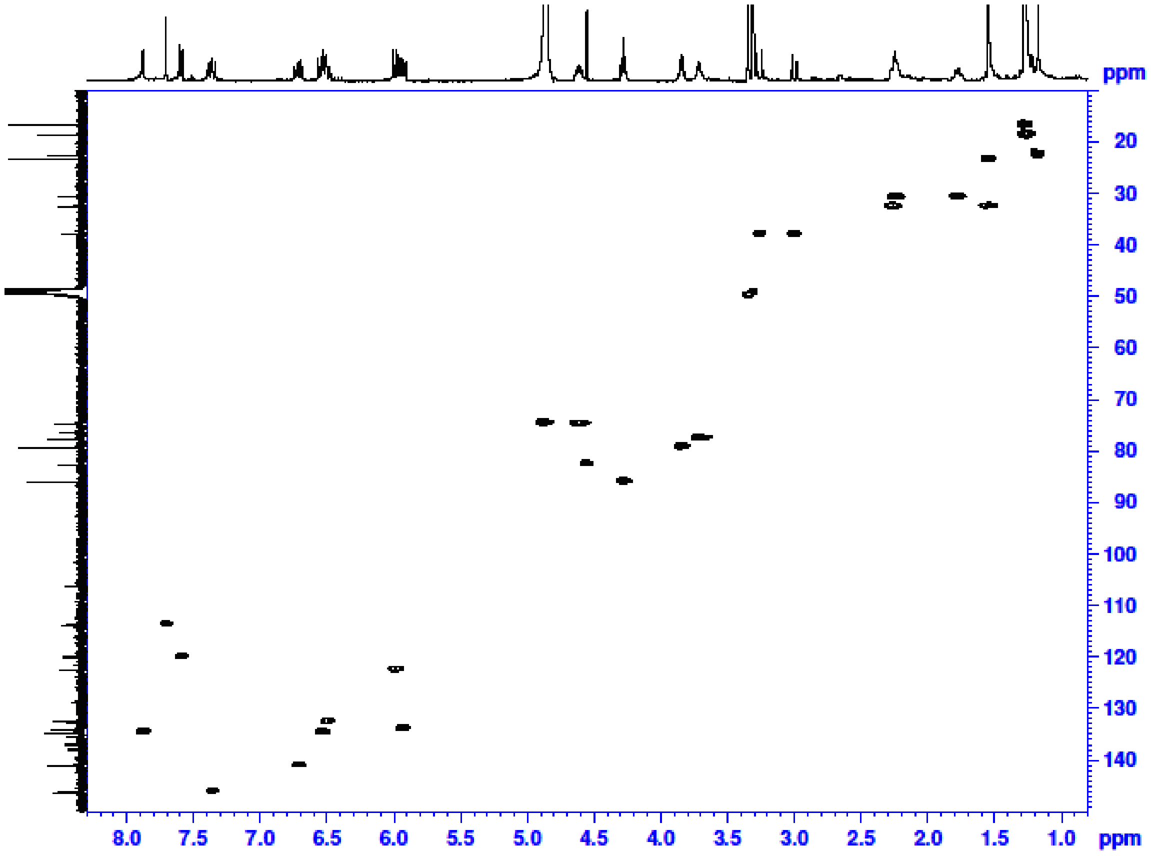

Figure S25. HMBC spectrum of **4** (in CD_3OD).

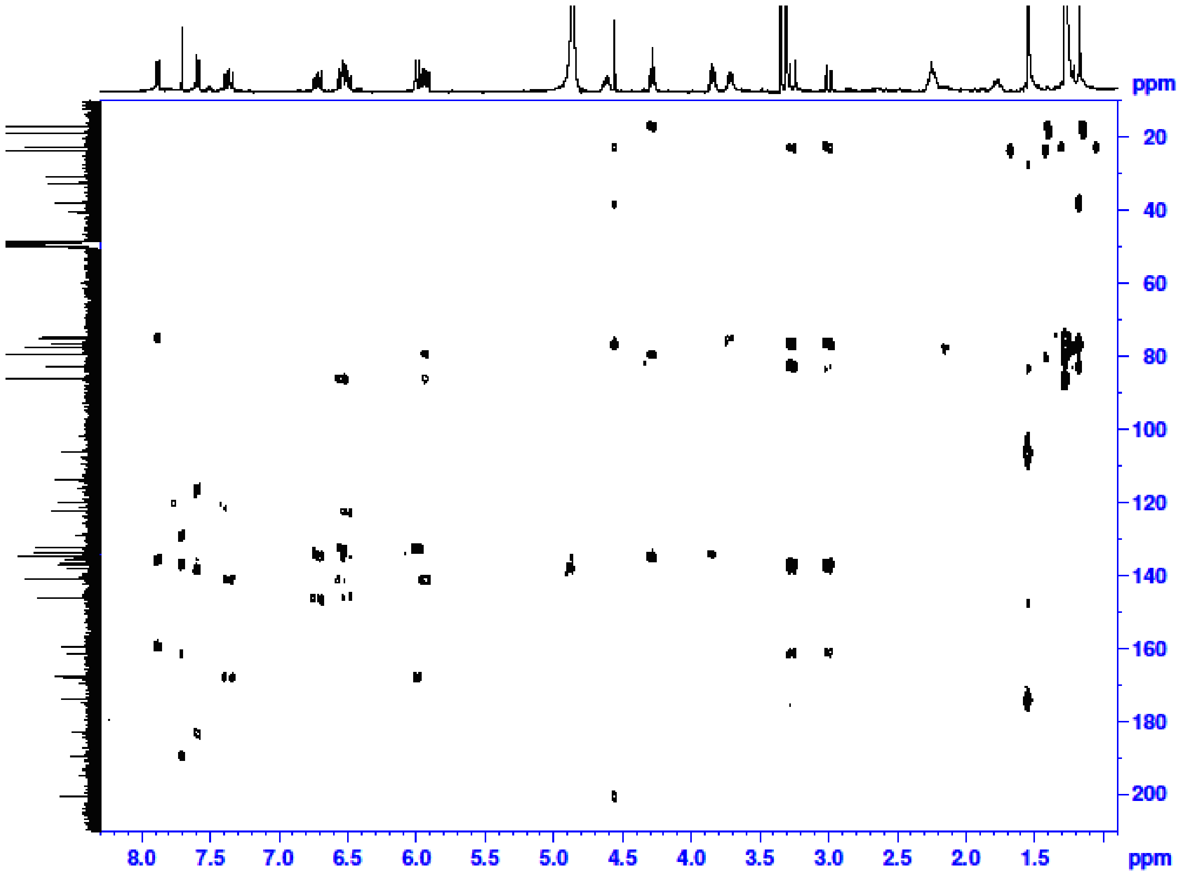

Figure S26. COSY spectrum of **4** (in CD_3OD).

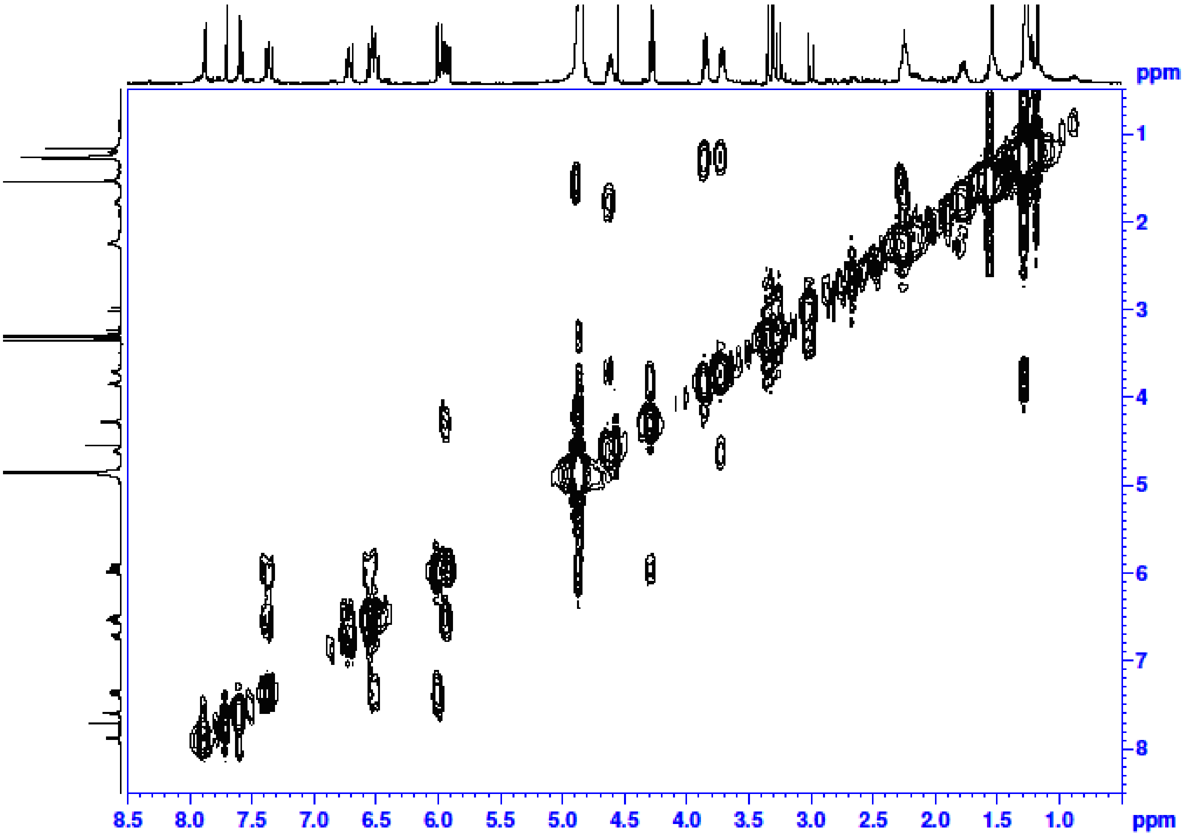

Figure S27. UV spectrum of **4**.

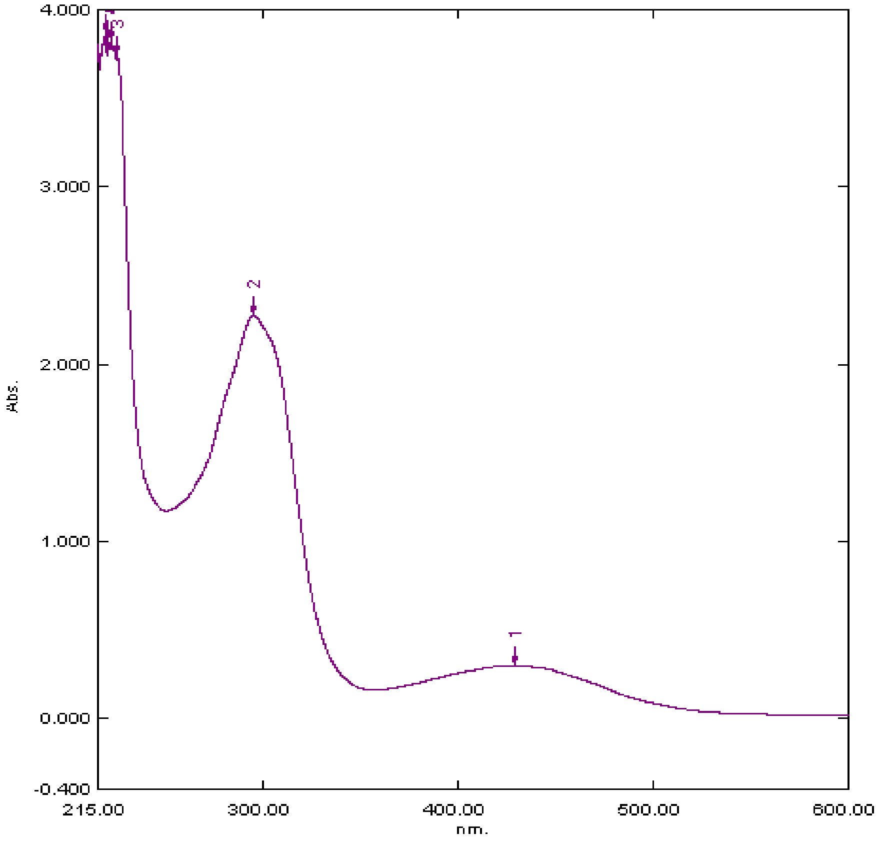

Figure S28. HRESIMS spectrum of **4**.

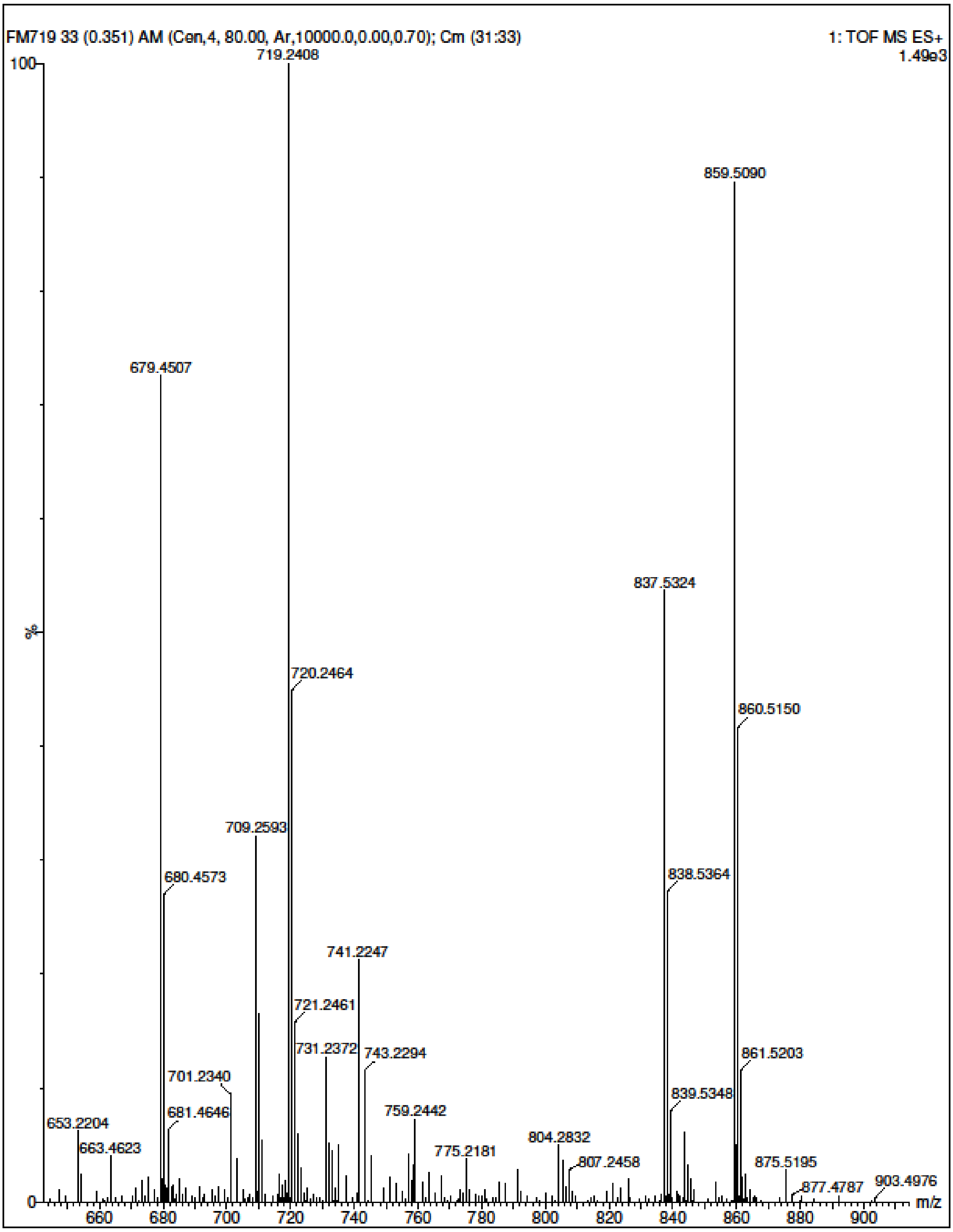

Figure S29. ^{1}H-NMR spectrum of **5** (in DMSO-d_6).

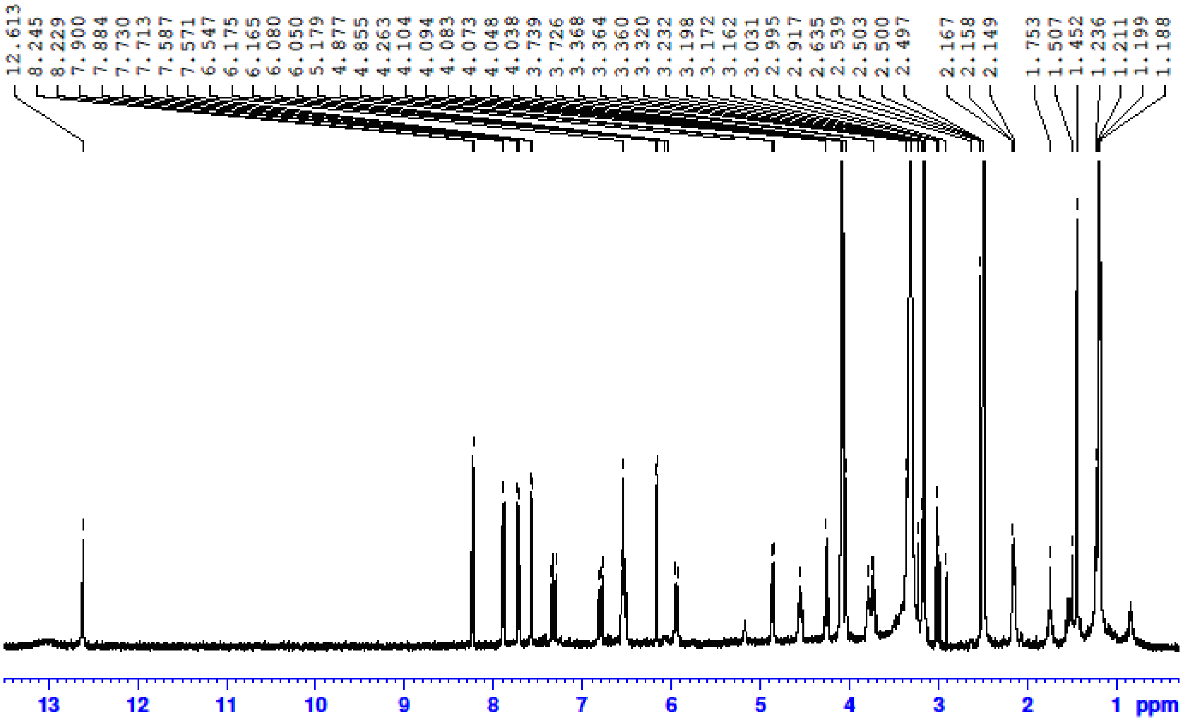

Figure S30. ^{1}H-NMR spectrum of **5** (in DMSO-d_6).

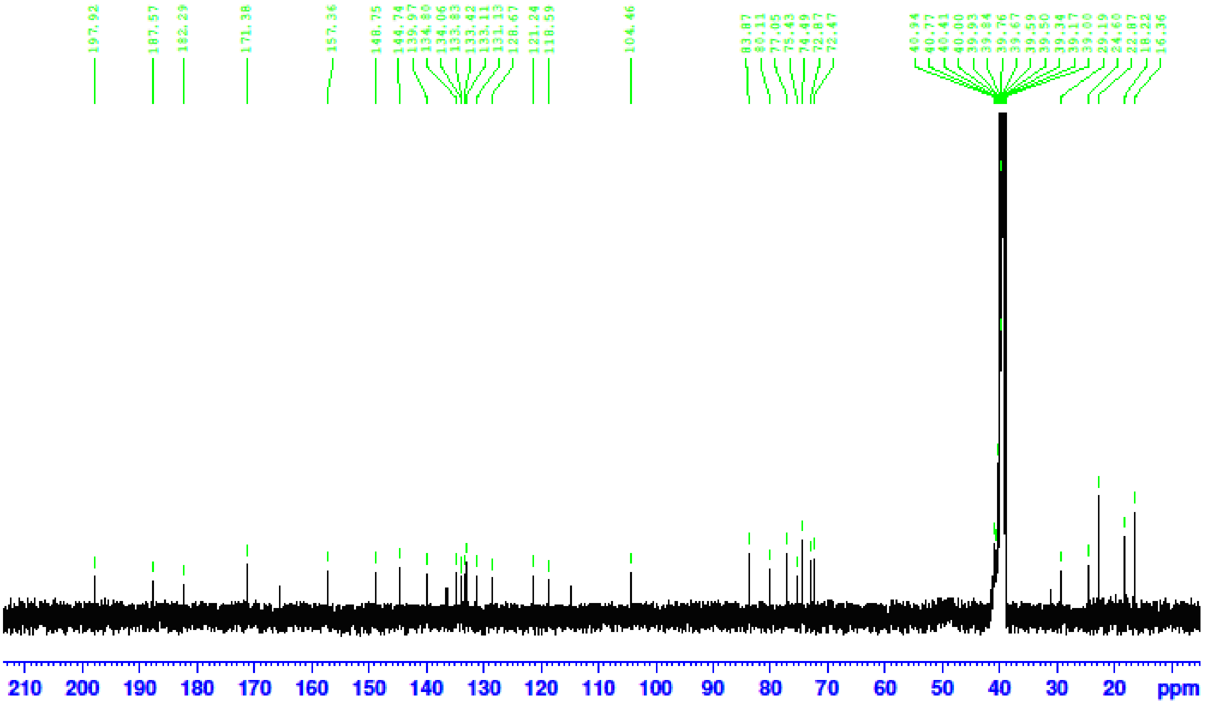

Figure S31. HMQC spectrum of **5** (in DMSO-d_6).

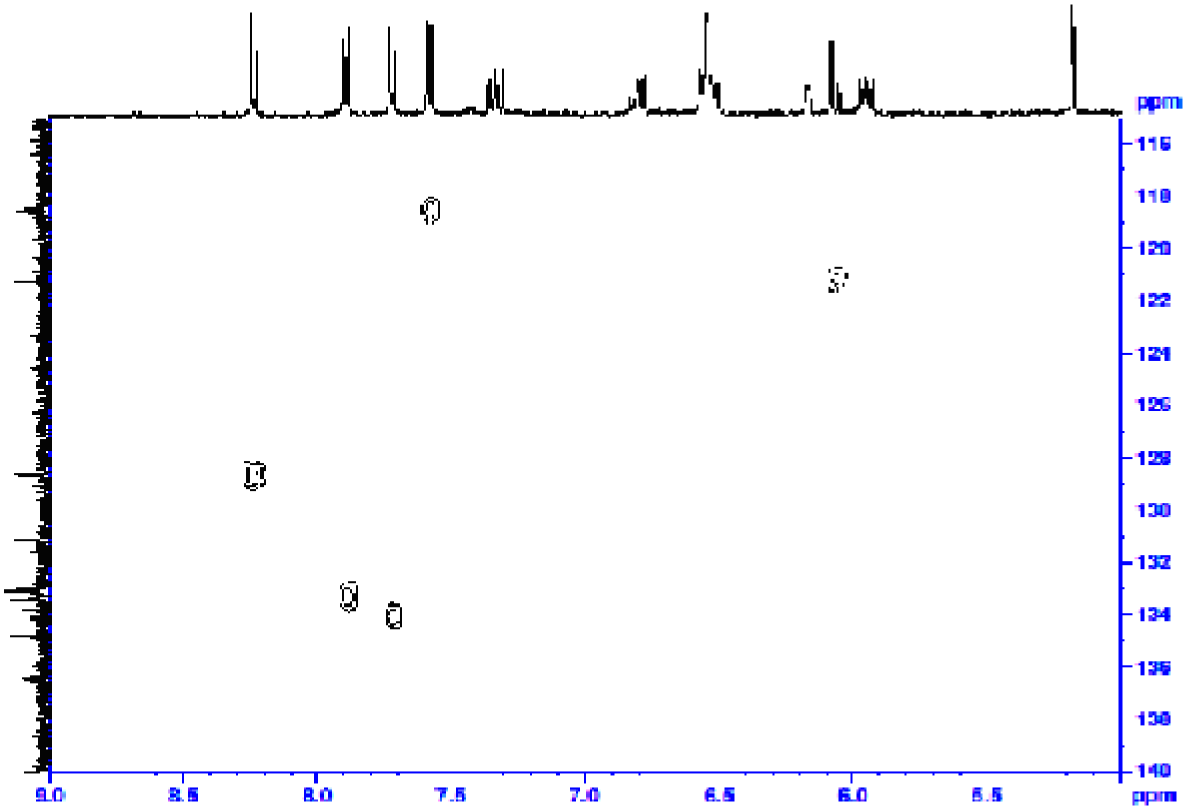

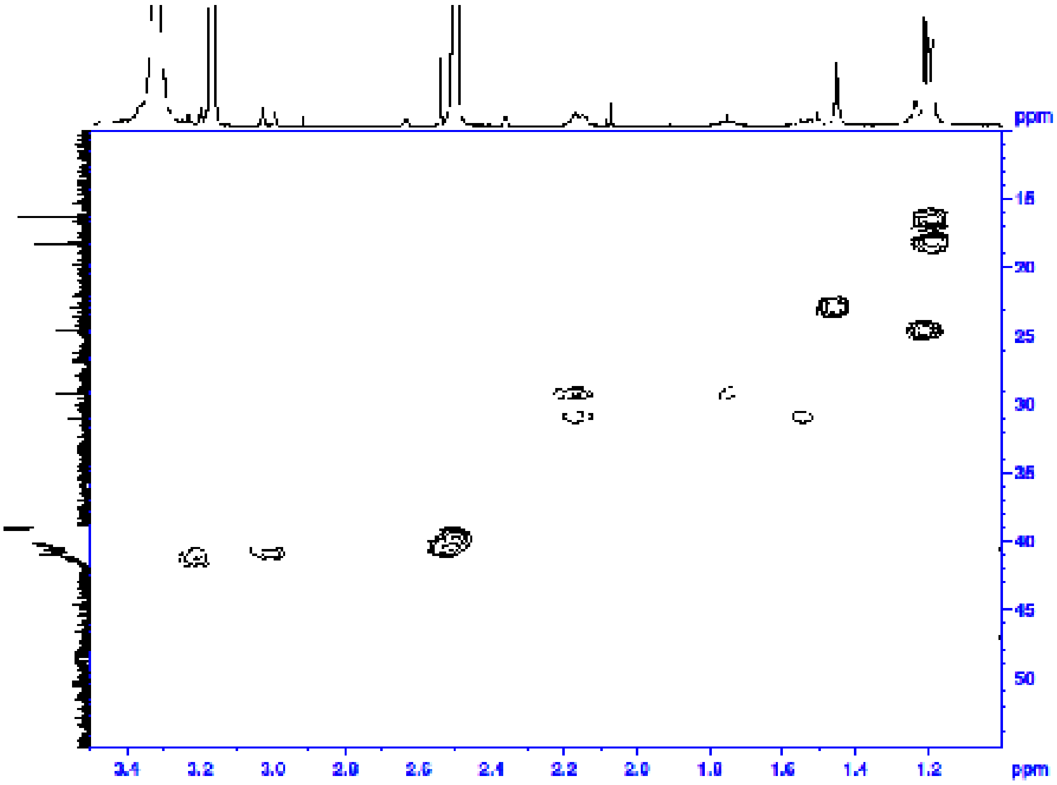

Figure S32. HRESIMS spectrum of **5**.

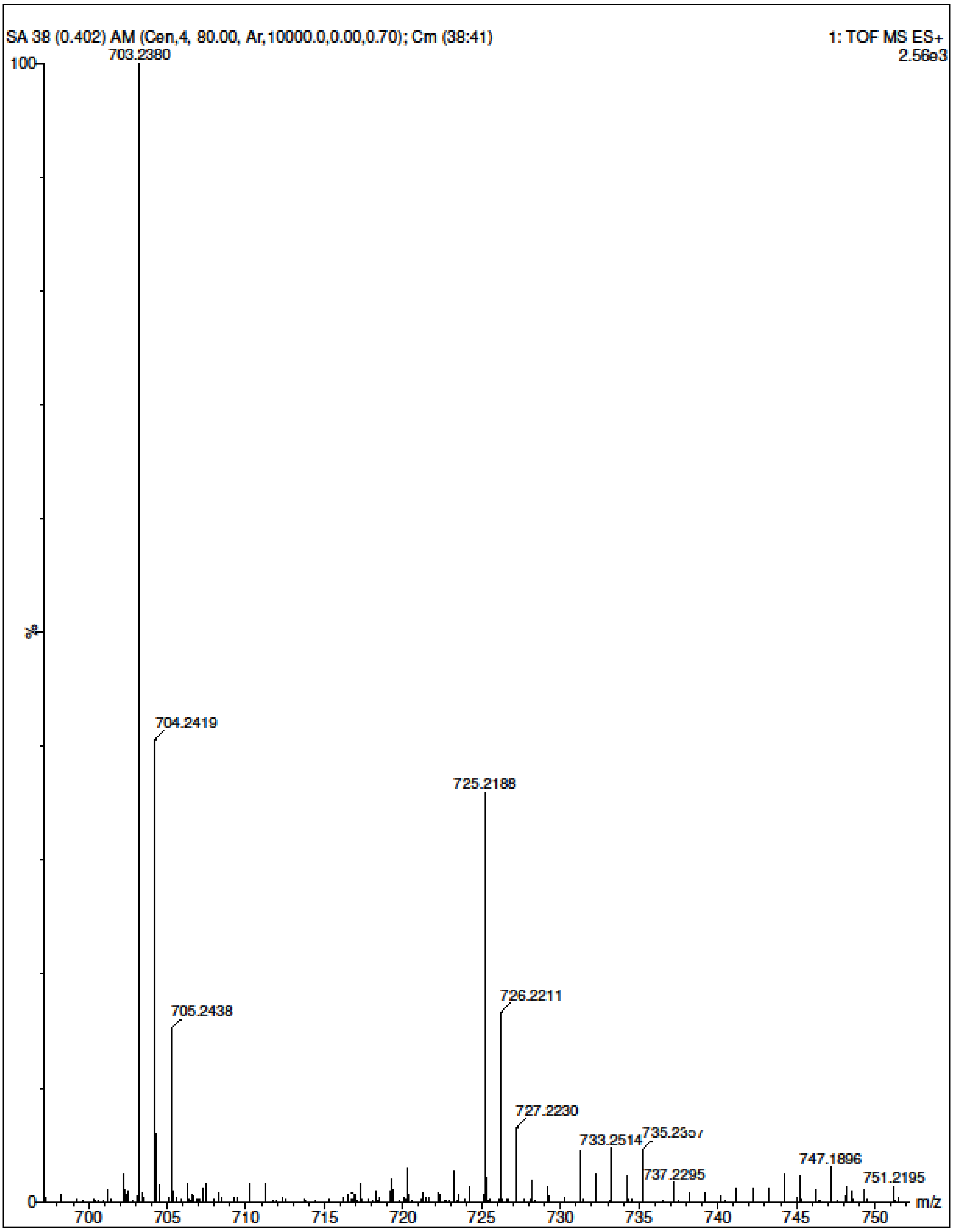

Figure S33. UV spectrum of **5**.

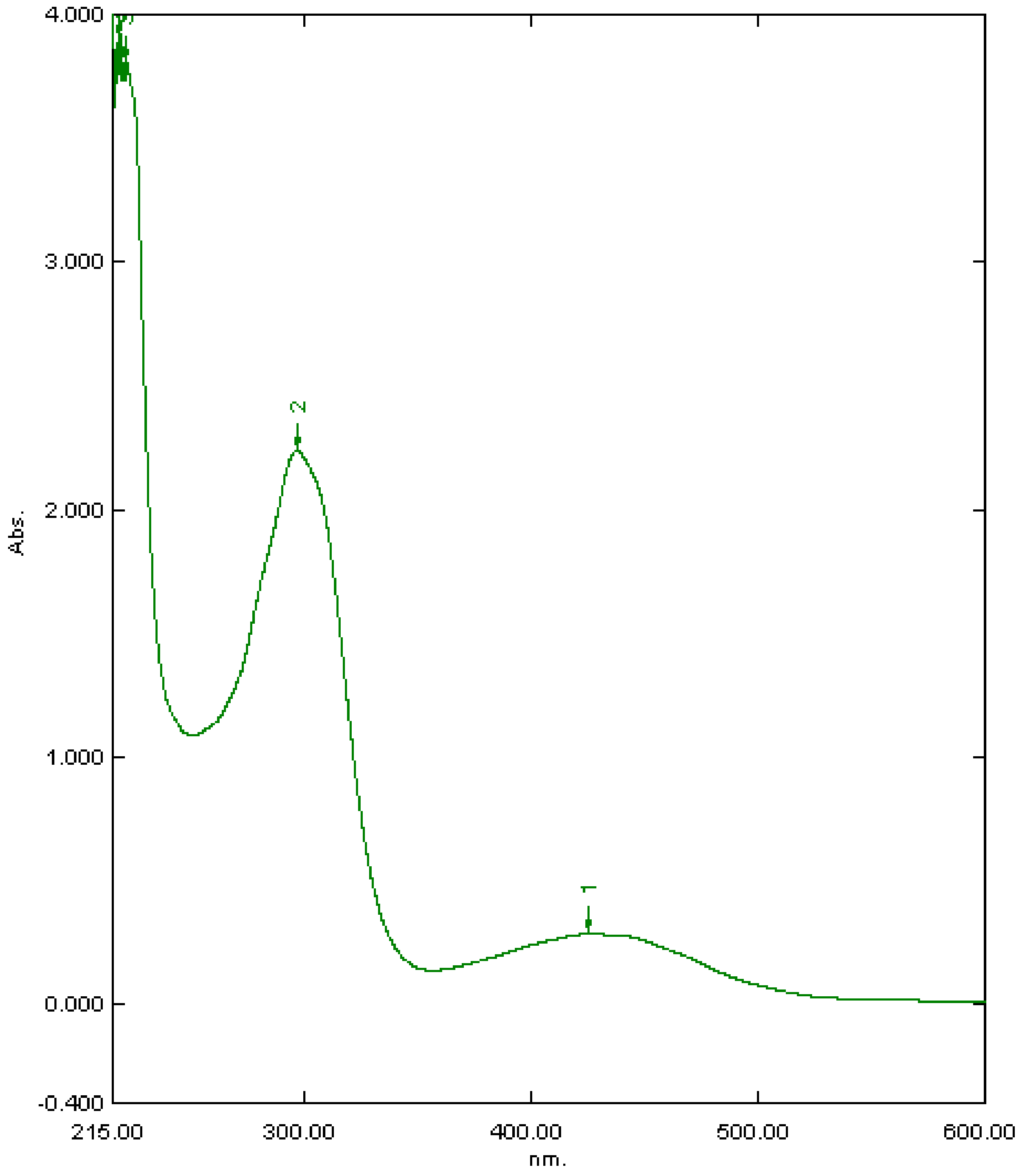

Figure S34. ^{1}H-NMR spectrum of **6** (in DMSO-d_6).

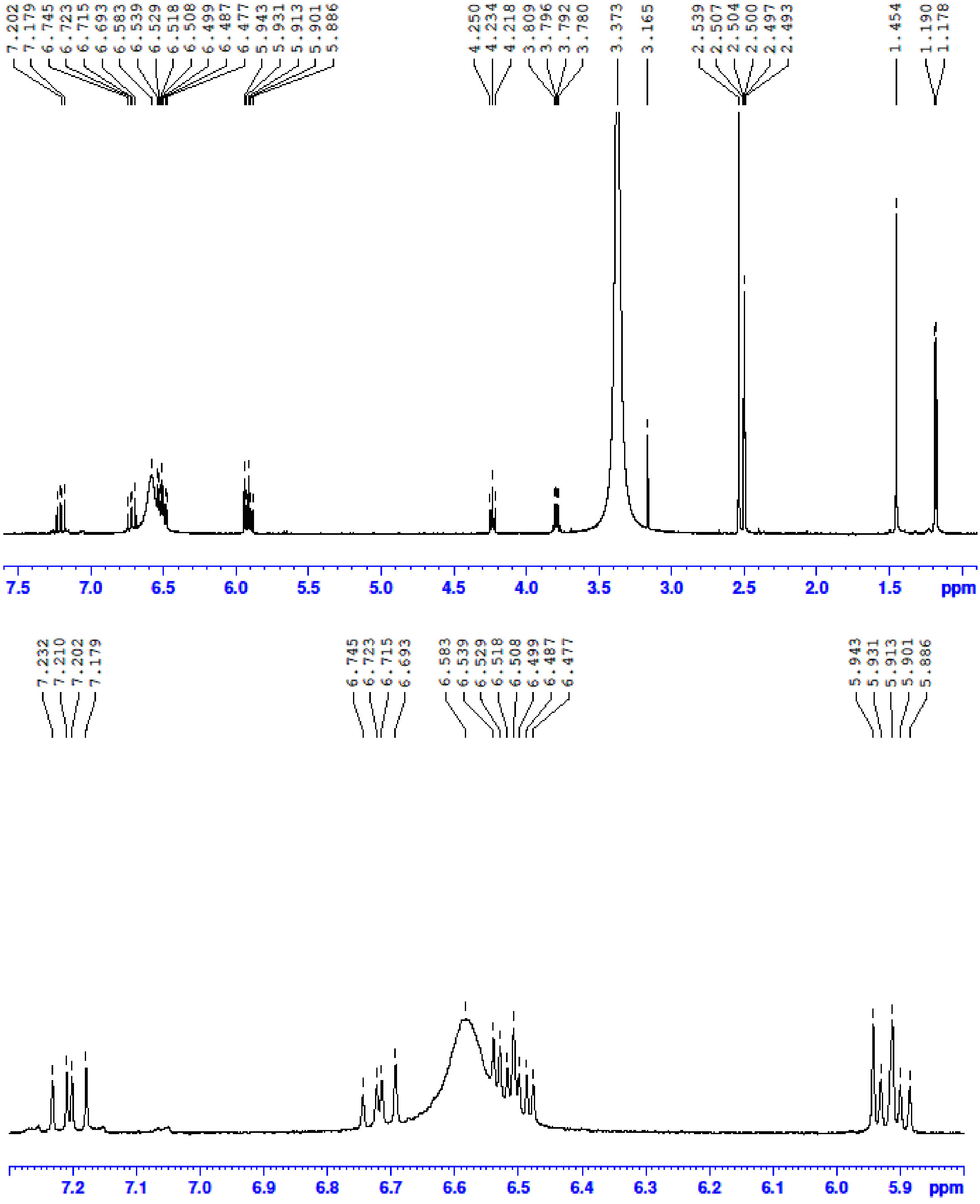

Figure S35. ^{13}C-NMR spectrum of **6** (in DMSO-d_6).

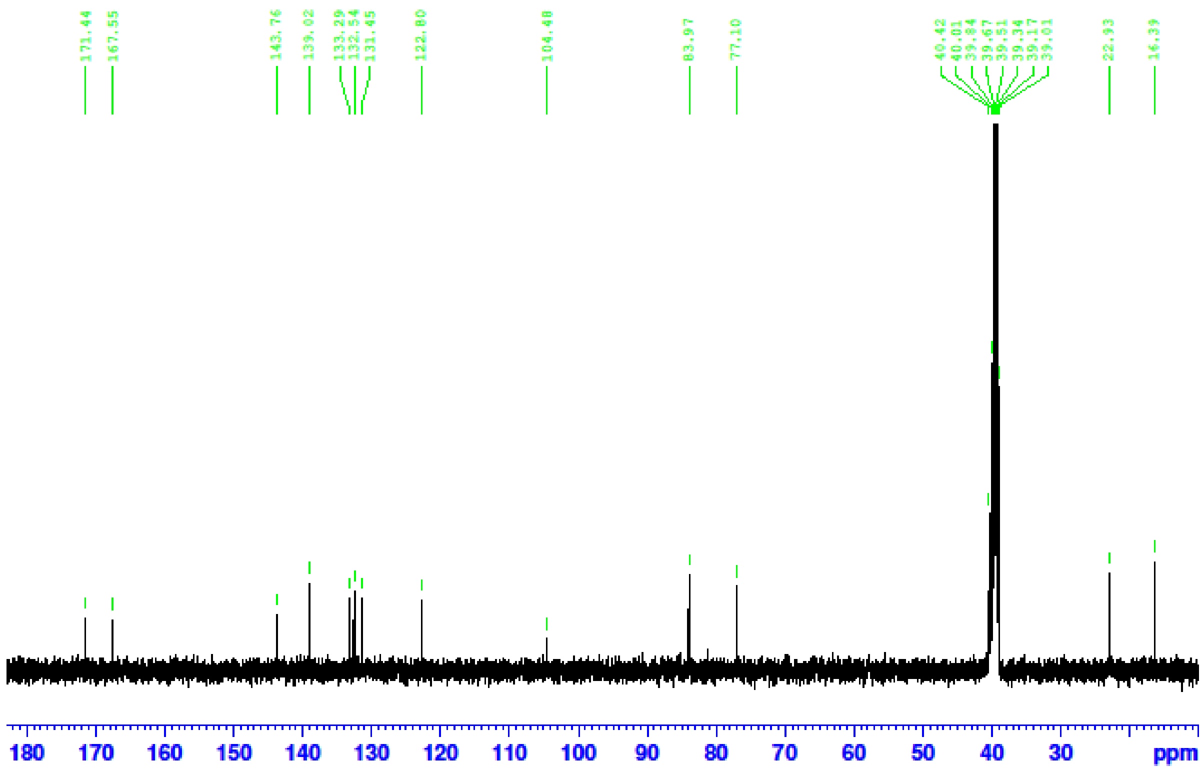

Figure S36. HMQC spectrum of **6** (in DMSO-d_6).

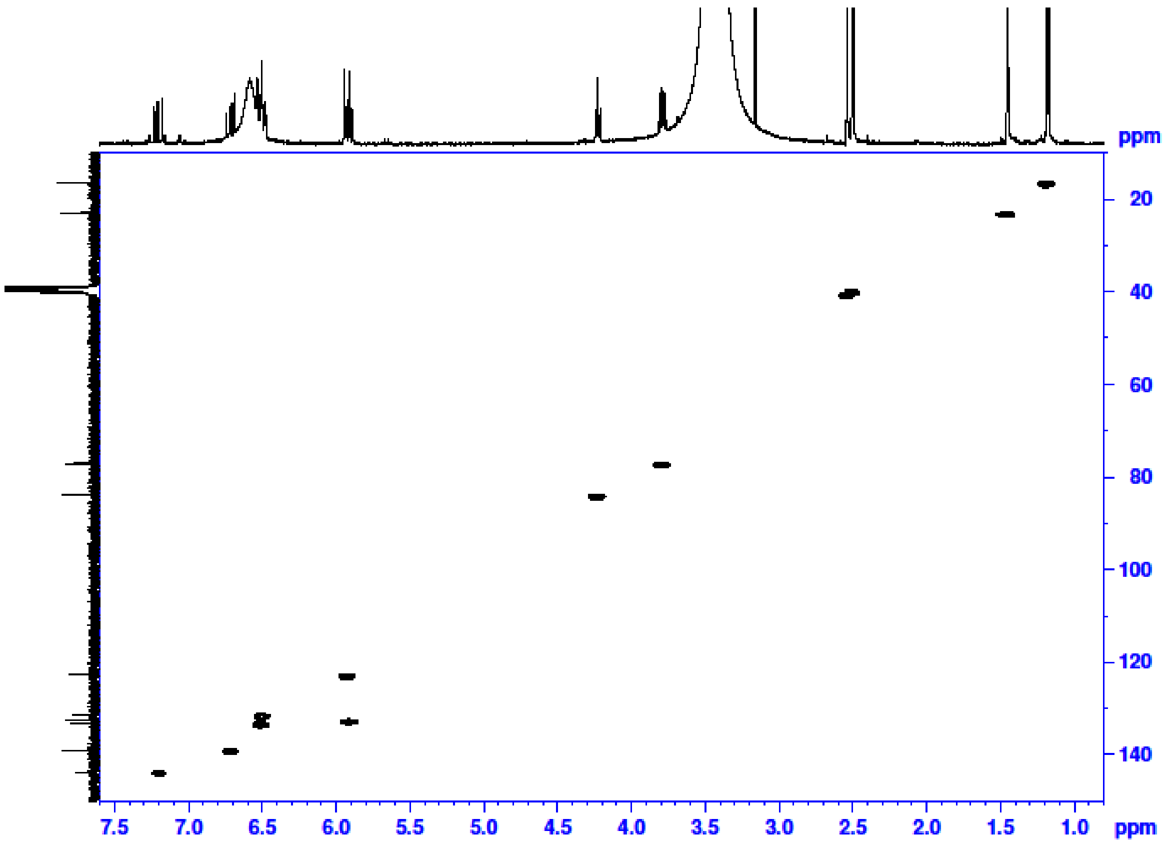

Figure S37. HMBC spectrum of **6** (in DMSO-d_6).

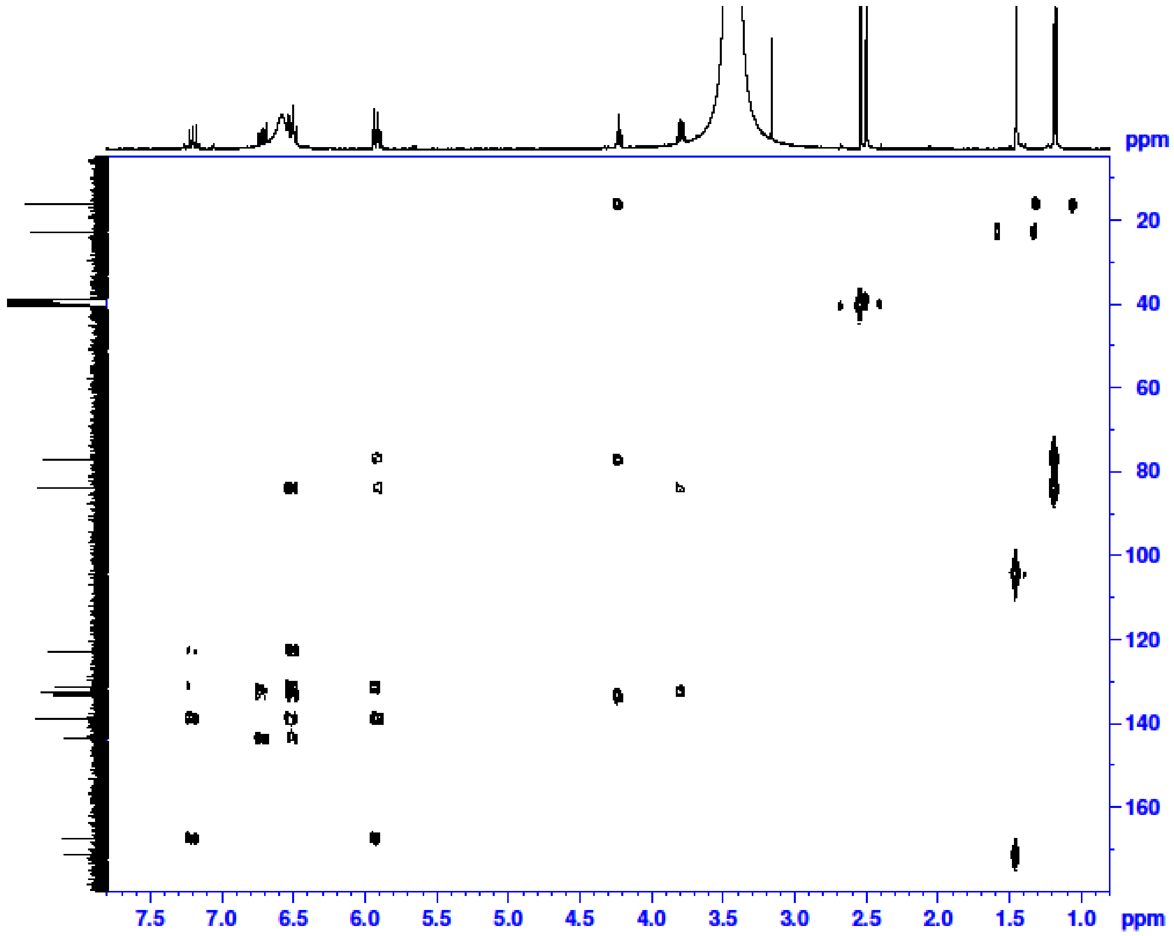

Figure S38. HRESIMS spectrum of **6**.

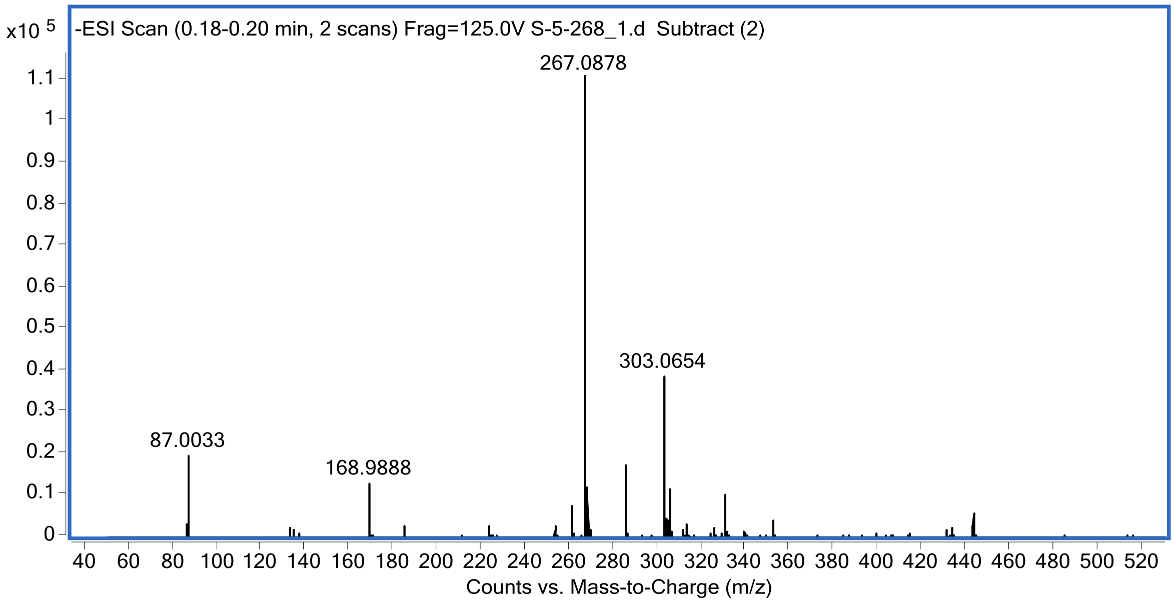

Figure S39. ^{1}H-NMR spectrum of **7** (in DMSO-d_6).

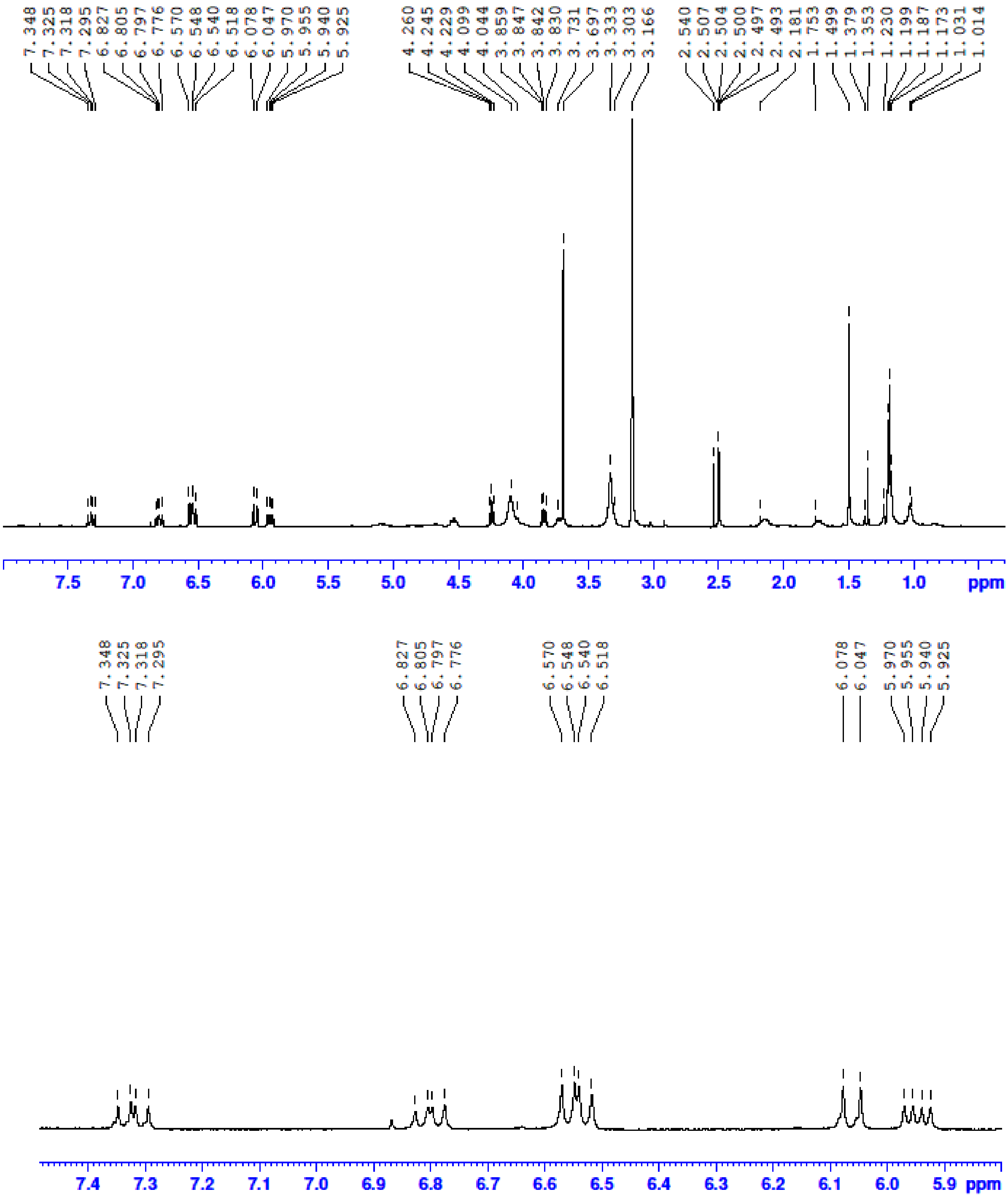

Figure S40. ^{13}C-NMR spectrum of **7** (in DMSO-d_6).

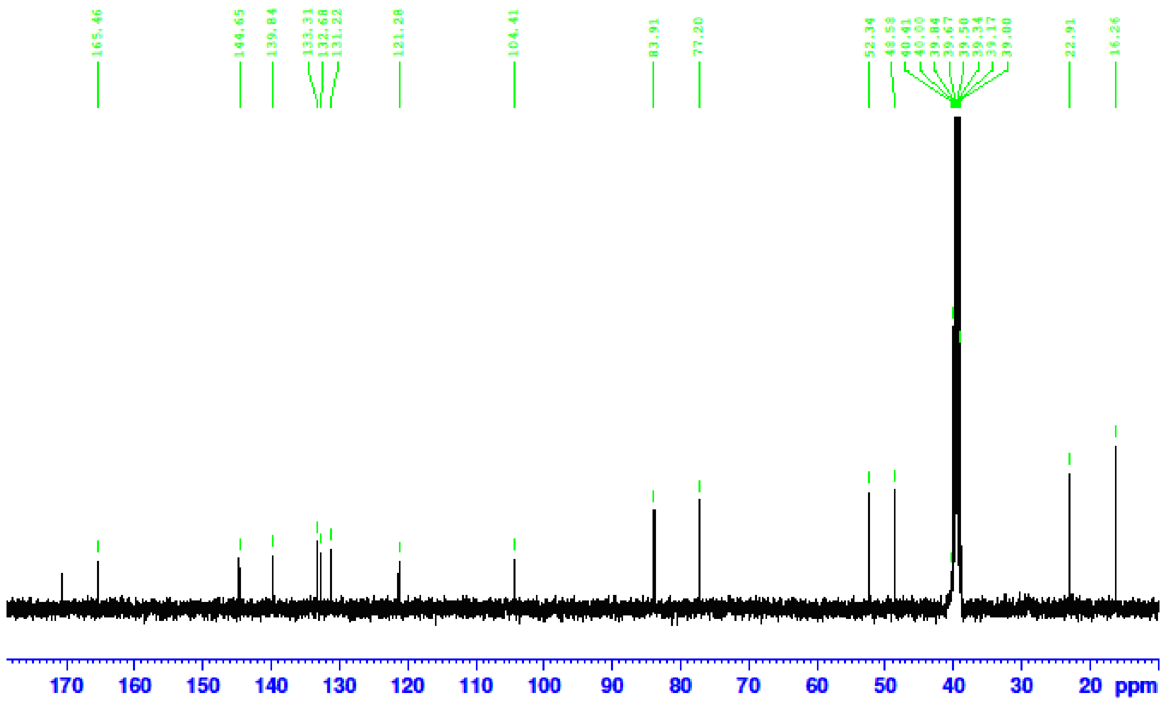

Figure S41. HMQC spectrum of **7** (in DMSO-d_6).

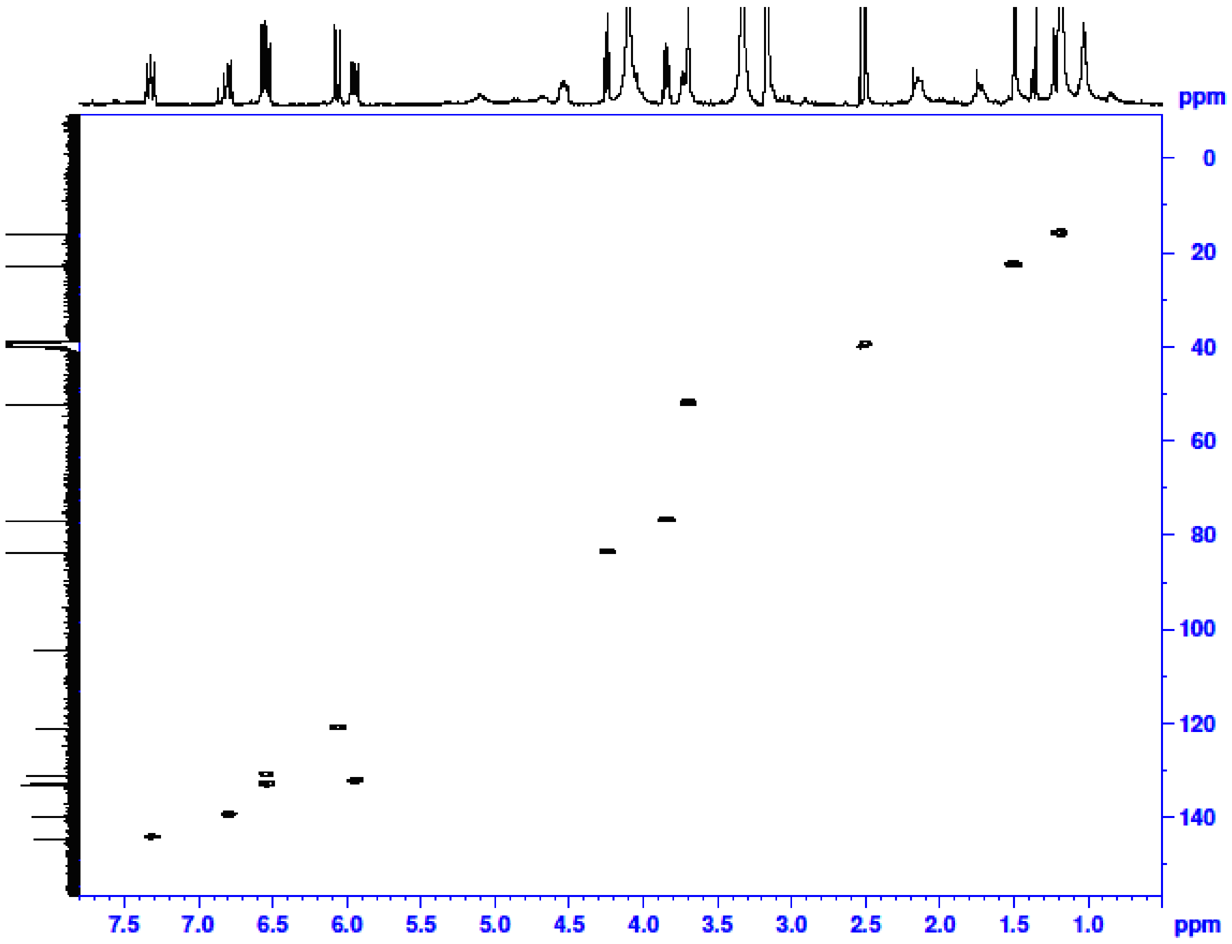

Figure S42. HRESI MS spectrum of **7**.

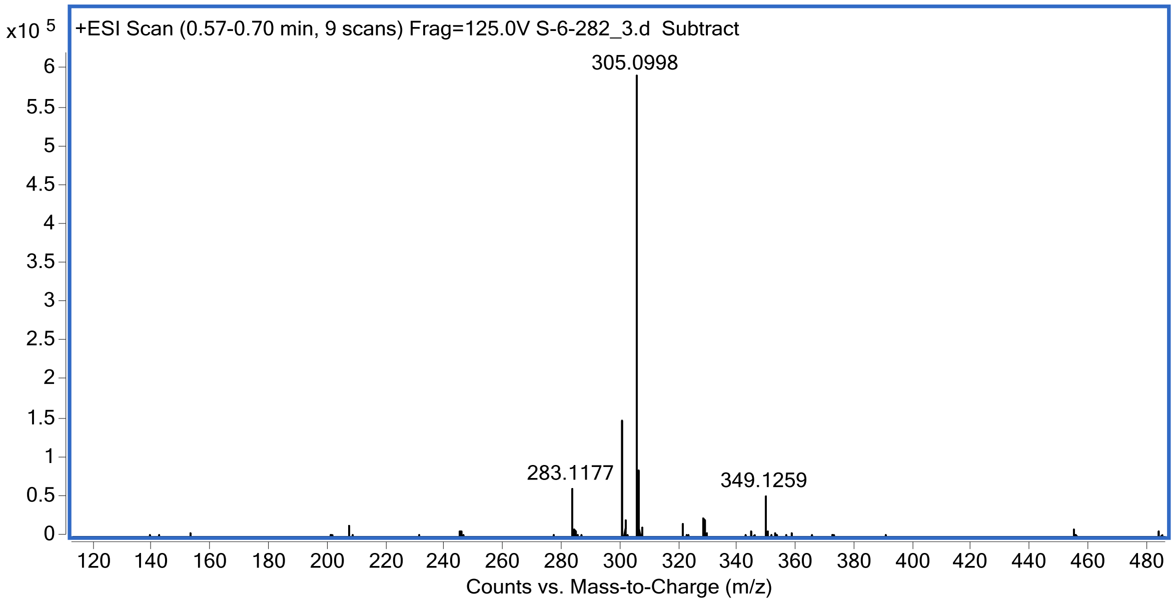

Figure S43. HPLC profiles of the ethanol extract (**A**) and the active 90% MeOH fraction (**B**).

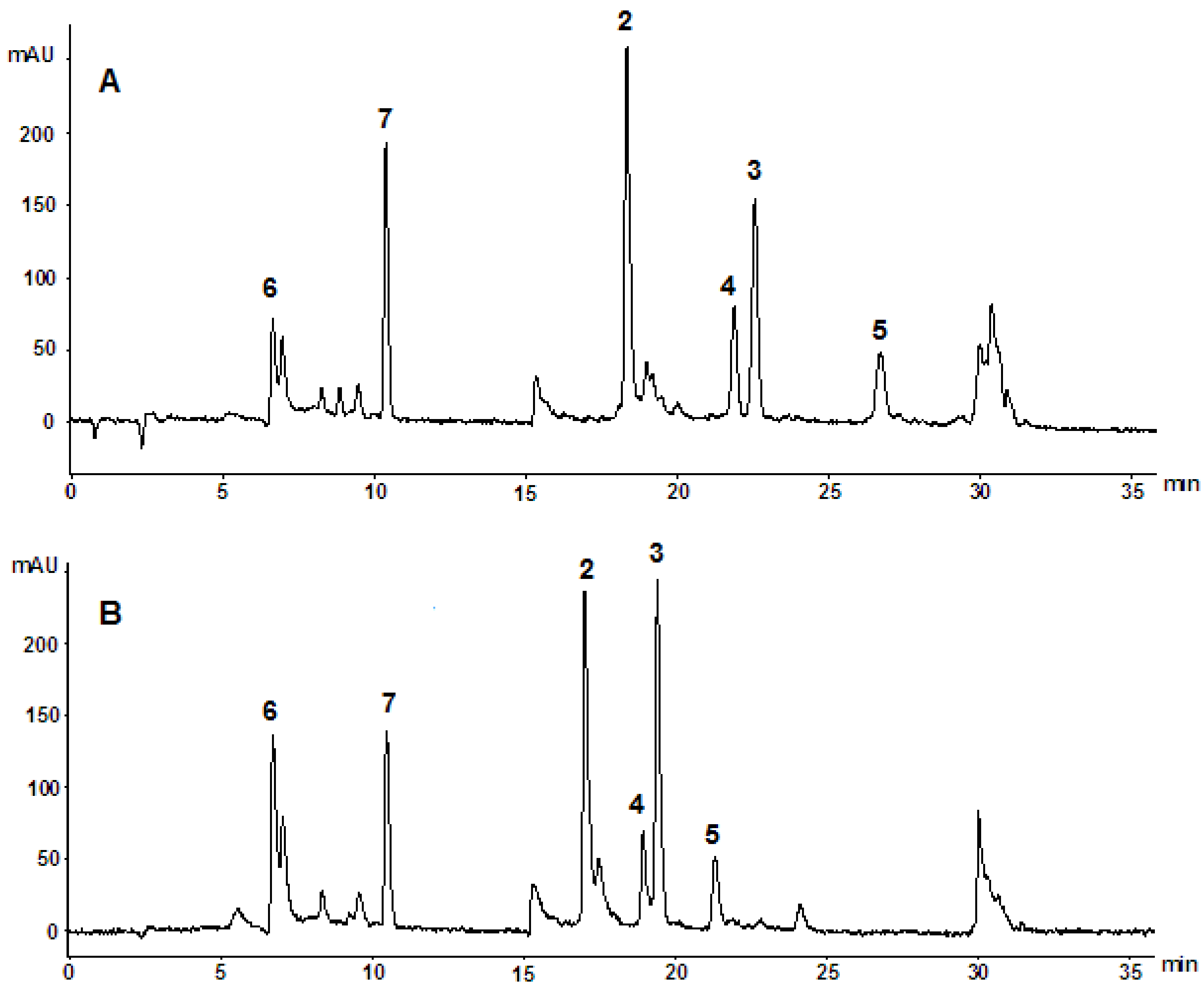

Figure S44. 16S rDNA sequence data of marine *Streptomyces fradiae* strain PTZ0025.

TGACGGGCGGTGTGTACAAGGCCCGGGAACGTATTCACCGCAGCAATGCTGATC
TGCGATTACTAGCGACTCCGACTTCATGGGGTCGAGTTGCAGACCCCAATCCGA
ACTGAGACCGGCTTTTTGAGATTCGCTCCACCTTGCGGTATCGCAGCTCTTTGTA
CCGGCCATTGTAGCACGTGTGCAGCCCAAGACATAAGGGGCATGATGACTTGAC
GTCGTCCCCACCTTCCTCCGAGTTGACCCCGGCGGTCTCCCGTGAGTCCCCAGC
ACCACAAGGGCCTGCTGGCAACACGGGACAAGGGTTGCGCTCGTTGCGGGACT
TAACCCAACATCTCACGACACGAGCTGACGACAGCCATGCACCACCTGTACACC
GACCACAAGGGGGCACCCATCTCTGGGTGTTTCCGGTGTATGTCAAGCCTTGGT
AAGGTTCTTCGCGTTGCGTCGAAtTAAGCCACATGCTCCGCCGCTTGTGCGGGC
CCCCGTCAATTCCTTTGAGTTTTAGCCTTGCGGCCGTACTCCCCAGGCGGGGAA
CTTAATGCGTTAGCTGCGGCACGGACGACGTGGAATGTCGCCCACACCTAGTTC
CCAACGTTTACGGCGTGGACTACCAGGGTATCTAATCCTGTTCGCTCCCCACGCT
TTCGCTCCTCAGCGTCAGTATCGGCCCAGAGATCCGCCTTCGCCACCGGTGTTC
CTCCTGATATMTGCGCATTTCACCGCTACACCAGGAATTCCGATCTCCCCTACCG
AACTCTAGCCTGCCCGTATCGAATGCAGACCCGGGGTTAAGCCCCGGGCTTTCA
CATCCGACGTGACAGGCCGCCTACGAGCTCTTTACGCCCAATAATTCCGGACAA
CGCTTGCGCCCTACGTATTACCGCGGCTGCTGGCACGTAGTTAGCCGGCGCTTCT
TCTGCAGGTACCGTCACTTTCGCTTCTTCCCTGCTGAAAGAGGTTTACAACCCG
AAGGCCGTCATCCCTCACGCGGCGTCGCTGCATCAGGCTTTCGCCCATTGTGCA
ATATTCCCCACTGCTGCCTCCCGTAGGAGTCTGGGCCGTGTCTCAGTCCCAGTGT
GGCCGGTCGCCCTCTCAGGCCGGCTACCCGTCGTCGCCTTGGTGAGCCGTTACC
TCACCAACTAGCTGATAGGCCGCGGGCTCATCCTGCACCGCCGGAGCTTTCCAC
CACCATCAGATGCCTGAAGTGGTCGTATCCGGTATTAGACCCCGTTTCCAGGGCT
TGTCCCAGAGTGCAGGGCAGATTGCCCACGTGT

Figure S45. Fradimycin B (4) induced apoptosis and necrosis in SW620 cells.

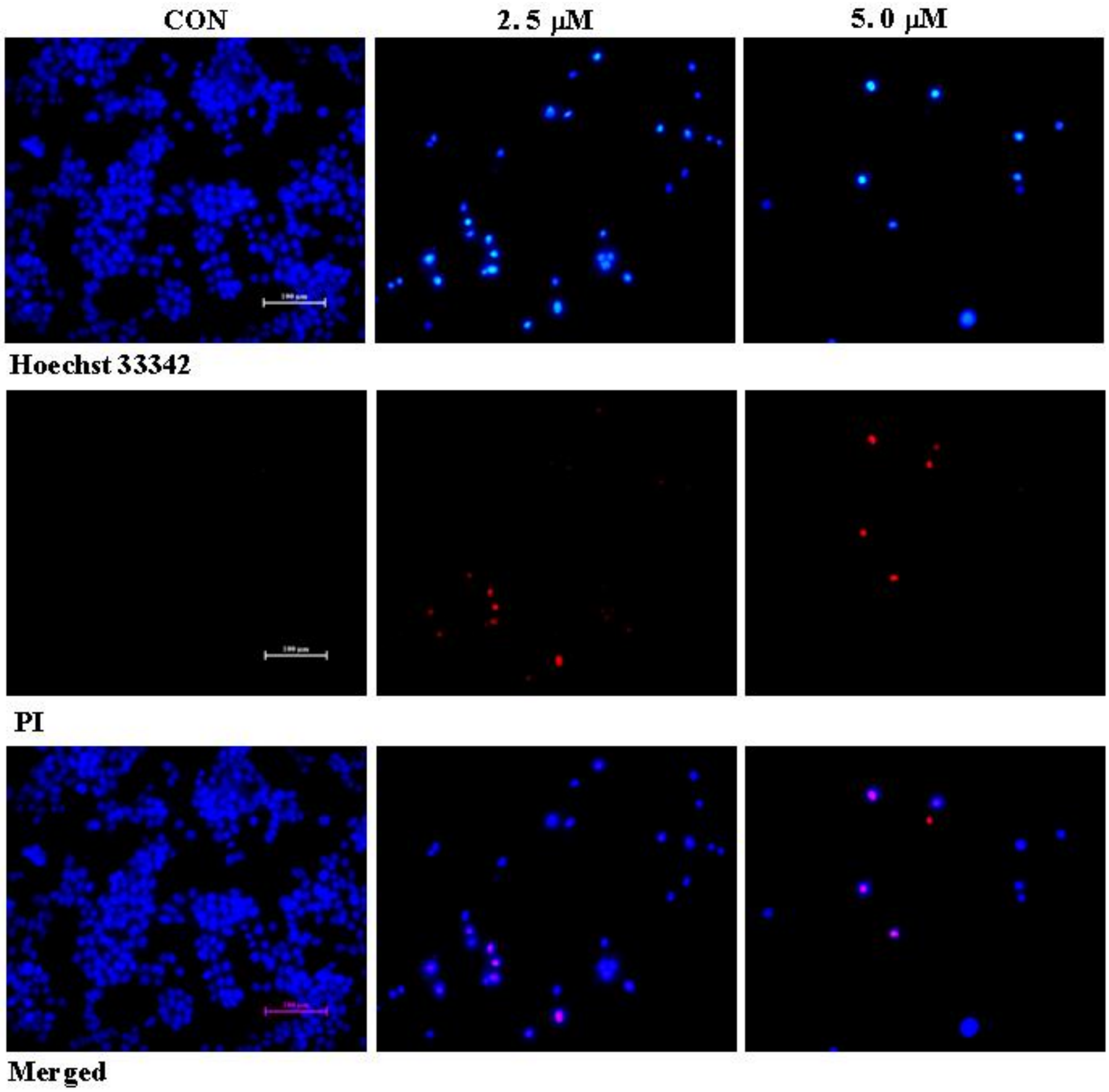

Figure S46. Fradimycin B (4) induced apoptosis and necrosis in rat glioma C6 cells.

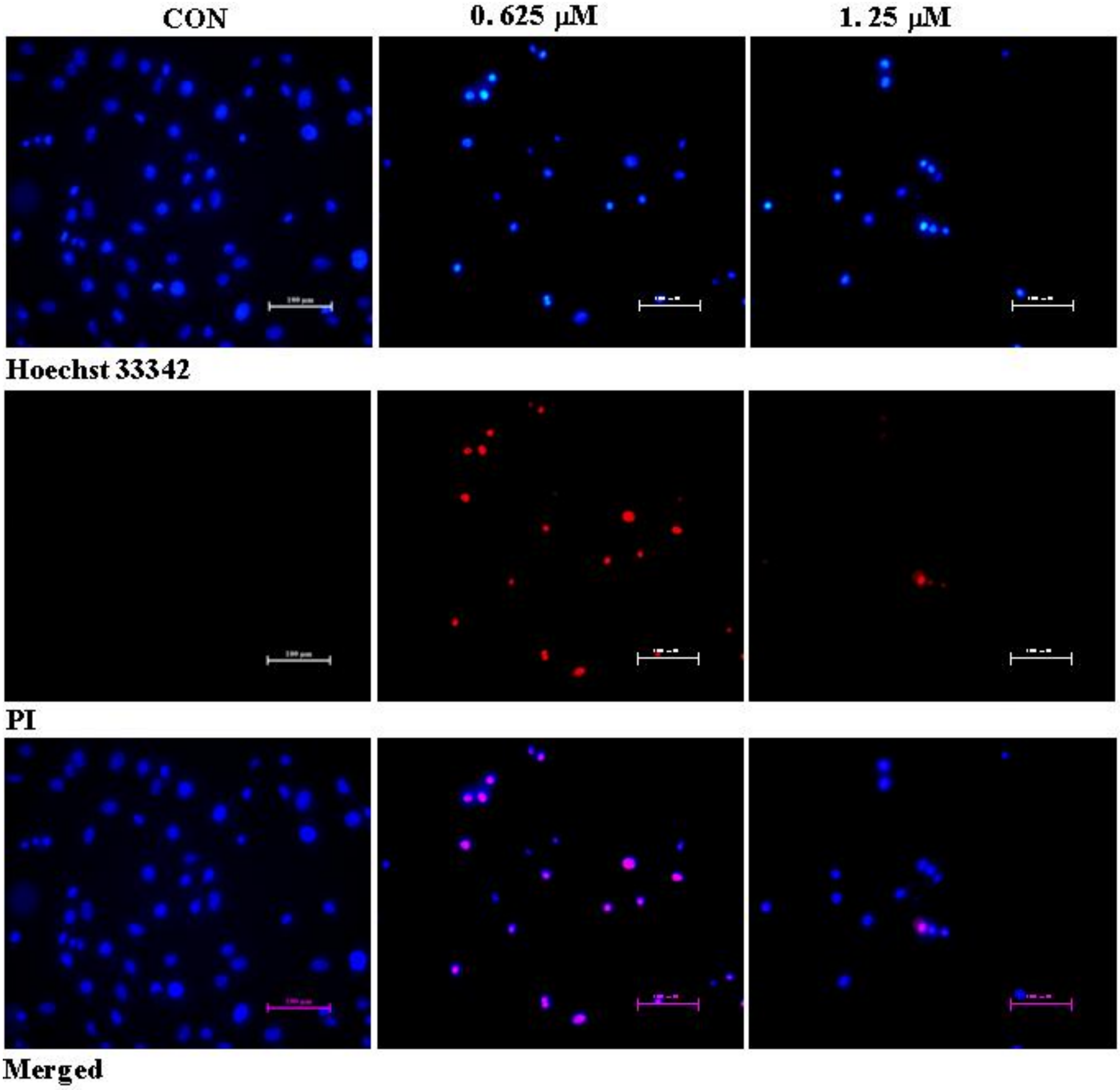

Supplement: Supplementary File 1: — PDF-Document (PDF, 768 KB) [file marinedrugs-10-02388-s001.pdf]
